# Exogenous SPD inhibits trastuzumab-mediated cardiomyocyte pyroptosis through SIRT3-regulated mitochondrial quality control

**DOI:** 10.7150/ijbs.110580

**Published:** 2025-06-09

**Authors:** Xue Yu, Yan Yang, Tianzuo Chen, Qianbing Wang, Zitong Wang, Xi Gao, Qianxue Wang, Jinxiang Guo, Yuqin Wang, Yajie Zhao, Shilin Wang, Wei Lu, Xing Luo, Tielei Gao, Jiayuan Kou, Hong Li, Liming Yang

**Affiliations:** 1 Department of Pathophysiology, School of Basic Medical Sciences, Harbin Medical University, Harbin 150000, China.; 2 Department of Cardiology, The Second Affiliated Hospital of Harbin Medical University, Harbin, China.; 3 National Key Laboratory of Frigid Zone Cardiovascular Diseases, Harbin, China.; 4 The Key Laboratory of Myocardial Ischemia, Chinese Ministry of Education, Harbin, China.; 5 Department of Pathology, The First Clinical Medical College of Shandong Second Medical University, Weifang people's Hospital, 151st Guangwen Road, Weifang 261100, China.; 6 Department of Forensic Medicine Harbin Medical University Harbin 150088, China.; 7 Department of Biochemistry and Molecular Biology, Harbin Medical University, Harbin 150000, China.

**Keywords:** trastuzumab, pyroptosis, mitochondrial biosynthesis, spermidine

## Abstract

Trastuzumab (TRZ) is an anti-HER2 monoclonal antibody associated with significant survival benefits; however, its clinical utility is restricted by trastuzumab-induced cardiotoxicity (TIC). While the inhibition of HER2 induces mitochondrial dysfunction in cardiomyocytes, it is unclear whether mitochondrial quality control participates in trastuzumab-mediated cardiomyocyte pyroptosis. This study demonstrated that TRZ leads to a reduction in left ventricular systolic function, myocardial pyroptosis, and mitochondrial oxidative stress; alterations in the mitochondrial membrane potential; changes in mitochondrial permeability; mitochondrial dysfunction; and a decrease in mitochondrial biosynthesis in the murine heart. Supplementation with exogenous spermidine inhibits myocardial oxidative stress and mitochondrial dysfunction, and promotes mitochondrial biosynthesis in mice, thereby protecting cardiac function. Additionally, SIRT3 plays a protective role in TRZ-induced myocardial injury. In SIRT3 knockout mice, TRZ-induced cardiac injury was exacerbated, and mitochondrial damage was aggravated. In conclusion, these findings reveal the pathogenic mechanism underlying trastuzumab-induced cardiomyopathy and suggest a novel therapeutic target for preventing cardiotoxicity in HER2+ breast cancer treatment.

## 1. Introduction

Oncology cardiology is an emerging and rapidly evolving interdisciplinary field with the goal of minimizing morbidity and mortality related to cardiovascular disease in oncology patients 1. The therapeutic landscape for numerous cancers has markedly transformed owing to the advent of effective cancer therapies. While antineoplastic therapy is life-saving, it can induce severe adverse cardiac effects, specifically cardiotoxicity, encompassing the direct cytotoxic effects of chemotherapy, cardiac systolic dysfunction, and myocardial ischemia. The European Society of Cardiology Guidelines for Oncology Cardiology 2022 Edition states that cancer therapy-related cardiovascular toxicity (CTR-CVT) focuses mainly on cardiomyopathy, heart failure, myocarditis, and arrhythmias 2. Currently, dexpropylenol, angiotensin-converting enzyme inhibitors, angiotensin II receptor antagonists, β-blockers, and sodium‒glucose cotransporter protein 2 inhibitors are recommended for the treatment of CTR-CVT in high-risk patients or those with prior high-dose anthracycline exposure. However, further mechanistic studies and novel preventive/therapeutic strategies are still needed.

Human EGF receptor (HER)2 receptor tyrosine kinases play crucial roles in cardiac development during embryogenesis and serve as survival factors for the adult myocardium 3. Despite TRZ being a highly effective treatment for breast cancer in patients with HER2, cardiac insufficiency has been identified as a possible side effect in approximately 3%-7% of patients treated with TRZ alone 4, 5. TRZ-induced cardiotoxicity (TIC) is typically reversible and, therefore, is classified as type II cardiotoxicity 6. However, previous studies have demonstrated that TRZ induces cardiomyocyte apoptosis, cellular breaks, and ultrastructural alterations, thus challenging the concept of TIC reversibility 7, 8. ErbB2 is present in cardiomyocytes and, in the presence of neuromodulin-1, activates downstream signaling pathways, including extracellular signal-regulated kinase (ERK) 1/2- mitogen-activated protein kinase (MAPK) and phosphatidylinositol-3-kinase (PI3K)-Akt. These pathways promote a cardioprotective program that regulates apoptosis, hypertrophic growth, mitotic growth, cell elongation, cell-cell adhesion, angiogenesis, and sensitivity to adrenergic signaling. Thus, disruption of these signaling pathways may be harmful to the heart. Recent studies have shown that animal models with cardiac-restricted ErbB2 knockouts display a dilated-type cardiomyopathy phenotype 9. Initially, there was a widespread belief that trastuzumab did not induce cardiac dysfunction. However, in a pivotal trial involving ErbB-2-positive metastatic breast cancer patients treated with trastuzumab, 27% of patients exhibited some degree of cardiac impairment, including an asymptomatic decrease in left ventricular systolic function, and symptomatic heart failure in 19% of patients 10. The inhibition of HER2 leads to mitochondrial dysfunction in cardiomyocytes 11. Mitochondrial dysfunction and changes in cardiac energy metabolism pathway were the main causes of cardiotoxic phenotypes induced by trastuzumab 12, 13. However, the specific molecular mechanism of trastuzumab-mediated cardiac damage remains unknown. Exploring these molecular mechanisms would offer new therapeutic strategies to minimize the myocardial toxicity associated with trastuzumab.

Initially demonstrated in 2001 to play a crucial role in the pathogenesis of cardiovascular disease, pyroptosis refers to the process of GSDM-mediated 12 programmed cell death (PCD) 14. GSDM can undergo cleavage, releasing the GSDM-N structural domain. This domain can perforate the cell membrane, inducing characteristic morphological changes, including cytoplasmic swelling, membrane rupture, and the release of inflammatory factors into the extracellular milieu. This process elicits a systemic immune response 15. Pyroptosis is characterized by heightened inflammation and the activation of caspase-1, caspase-11, and the NLR family containing the NLRP3 pyridine structural domain. This activation results in the cleavage of Gasdermin D (GSDMD) or GSDME, accompanied by plasma membrane rupture and the release of interleukin-1β (IL-1β) and IL-18 16. Numerous studies have demonstrated that doxorubicin mediates cardiomyocyte pyroptosis. However, it remains unknown whether trastuzumab induces cardiomyocyte pyroptosis.

Sirtuin 3 (SIRT3) is an NAD-dependent deacetylase implicated in a broad spectrum of physiological and pathological processes. Mammalian SIRT3 is predominantly located in mitochondria 17. SIRT3 plays a critical role in maintaining mitochondrial homeostasis, regulating metabolism, modulating gene transcription, responding to stress, and preserving genome stability 18. The primary functions of SIRT3 in mitochondria are to regulate the electron transport chain (ETC) to maintain basal ATP levels. In SIRT3 knockout mice, ATP levels were decreased by over 50% in the heart, kidney, and liver 19. SIRT3 promotes mitochondrial biosynthesis through AMPK activation. This activation either involves the direct phosphorylation of PGC-1α or enhances SIRT1-mediated PGC-1α activation 20, thereby promoting mitochondrial biosynthesis. Mitochondrial biosynthesis is crucial for maintaining mitochondrial integrity 21. Monitoring cellular mitochondrial integrity serves as a pivotal checkpoint in regulating programmed cell death and the release of inflammatory mediators. Altered mitochondrial homeostasis precipitates a paradigm shift in cell death 22. In addition, studies have shown that spermidine delays the senescence of multipotent mesenchymal cells through the antioxidant effect mediated by SIRT3 23.

Health- and life-span-promoting effects are documented for dietary or otherwise externally supplied spermidine (SPD). Spermidine supplementation confers life-span extension both to invertebrate model organisms 24 and to mice 25. Spermidine play diverse biological roles, including anti-inflammatory effects, inhibition of oxidative stress, participation in apoptosis, promotion of cell proliferation, modulation of ion channels, involvement in various cellular signaling pathways, and anti-aging effects. These molecules carry a strong positive charge at physiological pH and bind to cellular macromolecules, including proteins, nucleic acids, and phospholipid membranes, through acidic sites 26, 27. Naturally occurring polyamines possess the ability to scavenge free radicals, thereby reducing the level of oxidative stress in organisms and preventing oxidative damage to DNA and phospholipids in cell-free systems 28. Rider et al. reported that spermine plays a crucial role in protecting cells against H_2_O_2_-induced oxidative stress 29. Our previous studies showed that spermidine delayed aging in rats by promoting mitochondrial biosynthesis 30. Zheng Wei et al. demonstrated that exogenous polyamines alleviate inflammation-associated immune dysfunction 31. Jeong et al. found that spermidine significantly inhibited the production of pro-inflammatory cytokines, including nitric oxide and prostaglandin E2, as well as tumor necrosis factor α and IL-1β, in macrophages. Additionally, it attenuated lipopolysaccharide-induced inflammation 32.

Therefore, we propose the following scenario: Exogenous spermidine mitigates trastuzumab-induced myocardial injury by activating SIRT3. Consequently, SIRT3 promotes mitochondrial biosynthesis and diminishes oxidative stress.

## 2. Materials and Methods

### 2.1. Antibodies

The antibodies utilized in this study, along with details regarding their supplies, are provided in Table 1.

### 2.2. Animals

The Animal Protection and Use Committee of Harbin Medical University approved all animal experiments (Approval number HMUIRB2023033). Female C57BL/6J mice (aged 8 weeks), were purchased from the Liaoning Changsheng Biotechnology Co., LTD. Additionally, female SIRT3-KO C57BL/6J mice (aged 8 weeks), received as a friendly gift and genetically characterized 33, were acclimated for 1 week under standard animal husbandry conditions. Subsequently, wild-type C57BL/6J mice were randomly divided into 4 groups with 40 mice per group. Normal control group mice were intraperitoneally injected with normal saline for 3 weeks. Trastuzumab group was intraperitoneally injected with TRZ (10 mg/kg/3d) for seven times 34. The exogenous spermidine treatment group received intraperitoneal injection of trastuzumab and SPD at a dose of 10 mg/kg/ day for 3 weeks. In TRZ+SPD+DFMO group, mice in TRZ+SPD group were given 2% DFMO orally for 1 week and MGBG (15 mg/kg/d) intraperitoneally for 1 week. SIRT3-KO mice were randomly divided into 2 groups with 30 mice in each group. Mice in the control group were intraperitoneally injected with normal saline for 3 weeks, and the trastuzumab group was intraperitoneally injected with TRZ (10 mg/kg/3d) for a total of 7 times. At the conclusion of the treatment period, mice were anesthetized with avertin (200 mg/kg) and blood was taken from the eyeballs, and heart tissue samples were collected in liquid nitrogen for subsequent analysis.

### 2.3. Cell culture and treatment

The neonatal rat cardiomyocyte (NRCMs) and wistar fibroblasts, purchased from the Liaoning Changsheng Biotechnology Co., LTD, were isolated and cultured in DMEM high-glucose medium containing 10% fetal bovine serum for 48 hours. Subsequently, the cardiomyocytes were treated with 200 nM trastuzumab for 48 hours 35. Then, the cells were exposed to 10 µM SPD for 24 hours, and prior to these treatments, the cells underwent pretreatment with 100 µM AMPK agonist AICAR (MCE, HY-13417A) for two hours. Changes in the expression levels of various proteins were subsequently detected. Fibroblasts underwent treatment with 250 nM trastuzumab for 48 hours. Additionally, fibroblasts were treated with the antioxidant NAC (Sigma, A7250) at a concentration of 1 mM for 48 hours. Furthermore, fibroblasts were exposed to the mitochondrial scavenger of reactive oxygen species, Mito-tempo (Sigma, SML0737), at a concentration of 25 µM for 48 hours. Finally, the inhibitor of mitochondrial division, Mdivi-1 (MCE, HY- 15886), was applied to fibroblasts at a concentration of 20 µM for 48 hours. The HL-1 cardiomyocyte cell line was cultured in DMEM high-glucose medium containing 10% fetal bovine serum. Subsequently, the cardiomyocytes were treated with 200 nM trastuzumab for 48 hours, and 10 µM SPD for an additional 24 hours. Changes in the expression levels of various proteins were then detected. Additionally, interfering small RNA (control) was transfected into HL-1 cardiomyocytes. Furthermore, PGC-1α siRNA (Vizion Biologics) was utilized to interfere with gene expression. SIRT3 (VizenBio) was overexpressed in HL-1 cells through infection with adenovirus. Drug treatment was initiated 48 hours after infection.

### 2.4. Western blot

Treated HL-1 cells, NRCMs, fibroblasts, or ground myocardial tissue from treated mice, preserved in liquid nitrogen, were lysed with RIPA buffer on ice. Subsequently, the protein concentrations in the samples were assessed using the bicinchoninic acid (BCA) method with the BCA assay kit (Beyotime, P0010). In brief, proteins were separated on a 12.5% SDS-PAGE gel and subsequently transferred to a polyvinylidene difluoride membrane (Millipore, HATF, 2000). The blots of various antibodies were visualized using enhanced chemiluminescence (Yeasen, 36208ES60). The acquired images were subsequently analyzed using ImageJ software. Additionally, the expression levels of proteins in the samples were normalized to the expression level of GAPDH.

### 2.5. CO-IP immunocoprecipitation

Cell lysate was prepared, 4x107HL-1 myocardial cells were collected, washed with PBS, 1 ml RIPA buffer was added, thoroughly mixed, lysed on ice for 30 min, centrifuged at 12000 rpm at 4℃ for 10 min, and the supernatant was collected. 50 µl 50% Protein A/G Beads were added into the cell lysate, and the mixture was turned and incubated at 4℃ for 1 h for pre-elimination. After centrifugation, 0.5 ml supernatant was obtained, and 3 µg NRF-2 antibody was added. Another 0.5 ml supernatant was added with the same amount of homologous IgG antibody as the control. Shake and bind at 4℃ overnight, add 50µl 50% Protein A/G Beads to each tube on the second day, shake and bind at 4℃ for 3 h, and wash with RIPA Buffer 5 times for 5 min each time. After that, Western Blot analysis was performed. 25 µl 2×SDS loading Buffer was added to the precipitation, and the supernatant was centrifuged after boiling for 10 min. Western Blot analysis was performed with PGC-1α antibody.

### 2.6. RNA-seq and real-time PCR

Mice myocardial tissues were extracted and snap-frozen with liquid nitrogen. Subsequently, the samples were transported on dry ice to Beijing Biojingdian Biotechnology Co. Genes with a fold change of ≥2 were considered differentially expressed genes (DEGs). Subsequently, we employed Volcano plot analysis (https://cloud.oebiotech.cn/task/) to visualize the DEGs.

Myocardial tissue RNA was extracted using the Trizol method. The resulting RNA (2 µg) was then reverse transcribed into cDNA. For the qPCR, 0.4 µl of primer (10 µmol/µl) and 10 µl of SYBY-Green were added to 2 µl of cDNA, and the reaction was conducted using a Roche PCR system. The changes in the genes of each experimental group were subsequently calculated using the 2^-ΔΔCt^ method based on the resulting CT values. The sequences of the primers used are provided in Table 2.

### 2.7. Transmission electron microscopy

Extract 12 cubic millimeters of myocardial tissue from the left ventricle of mice in each experimental group. Subsequently, fix it with 2.5% glutaraldehyde phosphate buffer in a refrigerator at 4°C for 24 hours and prepare 60-80 nm slices after treatment. Observe the ultrastructural changes of myocardial tissue and mitochondria using a transmission electron microscope.

### 2.8. HE staining

Myocardial tissues were fixed in a 4% paraformaldehyde solution for 48 hours, dehydrated in alcohol, and embedded in wax blocks to create paraffin slices. Subsequently, the wax was dewaxed with various concentrations of xylene and ethanol. The slices were then washed with distilled water, stained with hematoxylin for 5 minutes, and rinsed with distilled water to remove the dye. Following this, the slices were stained with 0.5% eosin dye for 1 to 3 minutes, dehydrated with alcohol and clear xylene, and finally, sealed to observe the changes in the morphology and structure of the myocardium of the mice in each experimental group under a light microscope.

### 2.9. Masson staining

The left ventricular tissue of mice was taken and fixed in 4% paraformaldehyde at room temperature for 48 hours, then the tissue was embedded in paraffin wax and cut into 5µm thick tissue sections, dewaxed to water and stained with hematoxylin for 5 minutes, and washed with water. Then, stain it with the Masson-Pons acid fuchsin solution for 5 minutes, rinse thoroughly with distilled water; Phosphomolybdate aqueous solution was differentiated for 3 minutes, aniline blue solution was re-dyed for 5 minutes, soaked in 1% glacial acetic acid for a while, dehydrated and sealed, and microscope observed.

### 2.10. DCFH fluorescent probe

Cellular reactive oxygen species were detected using the DCFH assay kit (50101ES01, YEASEN). DCFH can permeate the chromosomal DNA and generate green fluorescence to quantify the amount of ROS. The DCFH working solution was diluted to a concentration of 10 µmol/L, and cardiomyocytes were added to the working solution for staining. They were then incubated at 37 ℃ for 30 minutes away from light, washed with PBS three times, and the generation of ROS in cardiomyocytes was observed using fluorescence microscopy.

### 2.11. JC-1 detection of mitochondrial membrane potential

Mitochondrial membrane potential was assessed using the Enhanced Mitochondrial Membrane Potential Assay Kit (C2003S, Beyotine). At a high mitochondrial membrane potential, JC-1 aggregates in the matrix of mitochondria, forming a polymer that produces red fluorescence. Conversely, at a low mitochondrial membrane potential, JC-1 cannot aggregate in the matrix of mitochondria, and in this case, JC-1 exists as a monomer, producing green fluorescence. Please refer to the instruction guide for the utilization of JC-1.

### 2.12. mPTP assay for mitochondrial membrane permeability

Mitochondrial membrane permeability was assessed using the Mitochondrial Permeability Transporter Pore Assay Kit (C2009S, Beyotine). The degree of mitochondrial mPTP opening was determined by assessing the strength of Calcein green fluorescence in mitochondria. A stronger green fluorescence indicates a lower degree of opening, while a weaker green fluorescence indicates a higher degree of opening. The Calcein AM working solution was diluted to a concentration of 10 µmol/L, and cardiomyocytes were added to the working solution for staining. They were then incubated at 37 ℃ for 30 minutes, protected from light, washed with PBS three times, and observed under fluorescence microscopy.

### 2.13. ATP activity assay

ATP levels in myocardial tissue were determined using the Enhanced ATP Assay Kit (S0027, Beyotine). 200 µl of lysate was added per 20 mg of myocardial tissue and homogenized using a glass homogenizer. Adequate homogenization ensures complete tissue lysis. Following lysis, the tissue is centrifuged at 12,000g for 5 minutes at 4°C. The supernatant is then removed, and 100 µl of ATP assay working solution is added to the assay wells. After 3-5 minutes, 20 µl of the sample or standard is added and rapidly mixed, with at least 2 seconds between mixings. The RLU value is then measured using a luminometer for subsequent analysis.

### 2.14. siRNA knockdown of PGC-1α

HL-1 cells were cultured in DMEM medium containing 10% fetal bovine serum as the control group (Scramble group). After 48 hours of culture, HL-1 cells in the normal group were cultured in DMEM medium under serum-free conditions. Subsequently, they were transfected with either Scramble or PGC-1α siRNAs using Lipofectamine 2000. Six hours after transfection, the HL-1 cells were cultured in DMEM medium containing 10% fetal bovine serum for an additional 24 hours, and cellular proteins were then extracted for subsequent experiments. Primer sequences are shown in Table 3.

### 2.15. Annexin V-FITC/PI cell scaling assay

Cellular focalization was assessed using the Annexin V-FITC/PI Cellular Focalization Assay Kit (40302ES60, YEASEN). PI, a nucleic acid dye, does not penetrate the intact cell membranes of normal or early apoptotic cells but does penetrate the cell membranes of late apoptotic and necrotic cells, staining the nuclei red. Therefore, when Annexin V is used in combination with PI, PI is excluded from living cells (Annexin V-/PI-) and early apoptotic cells (Annexin V+/PI-), whereas late apoptotic and necrotic cells stain double-positively (Annexin V+/PI+) with both FITC and PI. Please refer to the instruction manual for usage guidance.

### 2.16. Seahorse XF analyzer measurement for mitochondrial oxygen consumption rate (OCR)

HL-1 cells were seeded on XF-24-well culture microplates and allowed to differentiate for 7 days. After treatment, oxygen consumption was measured using a microplate (type XF24) extracellular analyzer from Seahorse Bioscience (Billerica, MA, USA). Reagents were optimized with the Mito Stress Kit from Seahorse Bioscience (Agilent, 100850-001) following the protocol and algorithm program in the analyzer. Following the measurement of the initial oxygen consumption rate (OCR), 1 µM oligomycin was added to inhibit ATP synthesis from oxidative phosphorylation. Subsequently, 1 µM carbonyl cyanide-4-(trifluoromethoxy)phenylhydrazone (FCCP) was introduced to uncouple the mitochondrial membrane, stimulating respiration. Finally, 1 µM rotenone and 1 µM antimycin A (R + A) were added to inhibit complex I and III, terminating mitochondrial oxidative phosphorylation.

### 2.17. Cells infected with adenovirus overexpressing SIRT3

Cellular SIRT3 overexpression adenovirus and negative control adenovirus were purchased from Shandong Weizhen Biological Company, and adenovirus transfection was performed according to the instructions. Primary mouse cardiomyocytes were homogeneously inoculated in cell culture dishes at a suitable density, and when the cell fusion rate reached 80%, the viral stock was diluted with DMEM culture medium until the multiplicity of infection (MOI) was 30, and the cells were incubated at 37℃ for 24 h. The cells were infected with adenovirus SIRT3, SIRT3-overexpressing adenovirus and negative control adenovirus.

### 2.18. Statistical analysis

All data in this study were expressed as mean ± standard deviation (Mean ± SD). The experiments were all 3 replicate independent experiments. Statistical comparisons were performed using one-way ANOVA followed by the S-N-K method multiple comparisons test, or the two-tailed Student's t-test using GraphPad Prism 9. Values of P < 0.05 were considered to be statistically significant (*, P < 0.05; **, P < 0.01; ***, P < 0.001).

## 3. Results

### 3.1. Exogenous spermidine treatment inhibits trastuzumab-mediated cardiac dysfunction in mice

**Figure 1:**
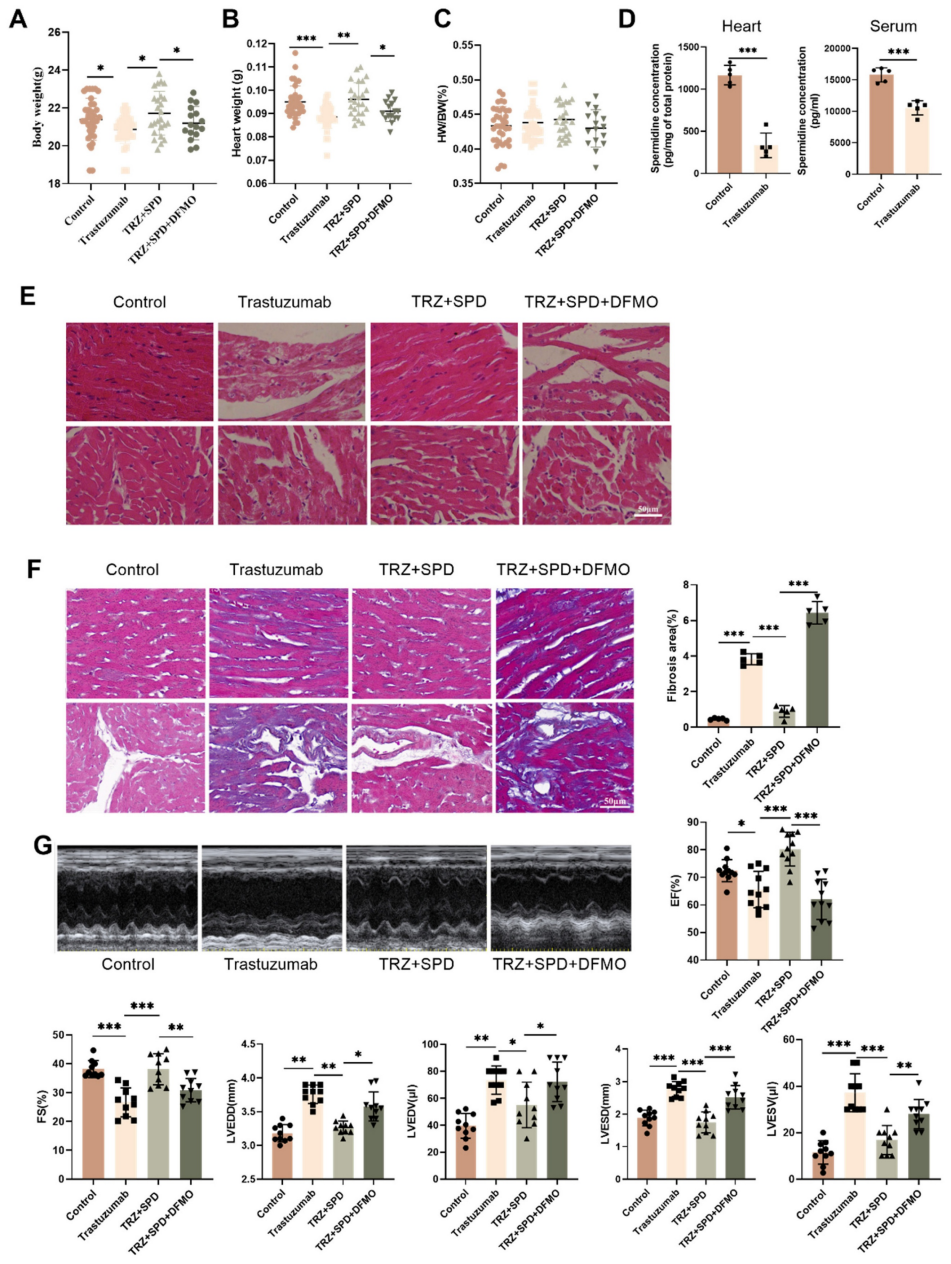
** Effects of trastuzumab on cardiac development in C57BL/6J mice.** (A) Body weight (BW), (B) heart weight (HW), and (C) heart weight to body weight ratio (HW/BW) of mice. Mean ± SD (n=35:37:25:16). *P < 0.05, **P < 0.01 and ***P < 0.001. *ANOVA*. (D) ELISA kits for the detection of trastuzumab-treated spermine in mouse heart and serum. Mean ± SD (n=5). ***P < 0.001. *t test*. (E) Representative HE staining of ventricular slices of each group (n=5), scale bar = 50 µm. (F) Representative Masson staining of ventricular slices of each group (n=5), scale bar = 50 µm. Mean ± SD. ***P < 0.001.* ANOVA*. (G) Echocardiographic detection of cardiac function in each group of mice. Ejection fraction (EF) (n=11), short-axis shortening (FS) (n=10), left ventricular end-diastolic internal diameter (LVEDD) (n=10), left ventricular end-diastolic volume (LVEDV) (n=10), left ventricular end-systolic internal diameter (LVESD) (n=10), left ventricular end-systolic volume (LVESV) (n=10). Mean ± SD. *P < 0.05, **P < 0.01 and ***P < 0.001. ANOVA.

We investigated the effects of trastuzumab on heart weight body weight and cardiac function in mice. Trastuzumab-administered C57BL/6J mice exhibited significantly lower body weight and heart weight, a phenomenon reversed by exogenous spermidine intervention (Fig. 1A-Fig. 1B). The heart weight to body weight ratio of the mice was not significantly altered (Fig. 1C). We examined the cardiac and serum levels of spermidine in trastuzumab-treated mice and found that the levels of spermidine were significantly reduced after trastuzumab treatment, so we supplemented exogenous spermidine as an intervention (Fig. 1D). Cardiac H&E staining showed that trastuzumab group mice had loose and disorganized myocardial structure, whereas TRZ+SPD group mice had well-arranged myofilaments and homogeneous cytoplasmic staining, but the effect of SPD was abolished in TRZ+SPD+DFMO group, and the myocardial structure of the mice was loose and disorganized (Fig. 1E). Meanwhile, Masson staining results showed that compared with the control group, trastuzumab group mice myocardial tissue collagen deposition was improved, both interstitial and perivascular, and the level of fibrosis was reduced (Fig. 1F). Conversely, in the exogenous spermidine intervention group, cardiac fibers were aligned, and there was no significant proliferation of connective tissues (Fig. 1E-Fig. 1F). Cardiac ultrasound results indicated that exogenous spermidine intervention significantly inhibited the trastuzumab-induced reduction in cardiac ejection fraction (EF) and short-axis shortening (FS), as well as the significant elevation of LVEDD, LVESD, LVEDV, and LVESV in mice. Notably, the addition of DFMO, a synthetic inhibitor of SPD, nullified the effect of exogenous spermidine (Fig. 1G). These results suggest that trastuzumab induces cardiac weight and body weight loss, decreased cardiac function and myocardial fibrosis in mice, and that exogenous spermidine intervention reverses these changes.

### 3.2. Exogenous spermidine treatment reduces trastuzumab-induced cardiomyocyte pyroptosis in mice

**Figure 2:**
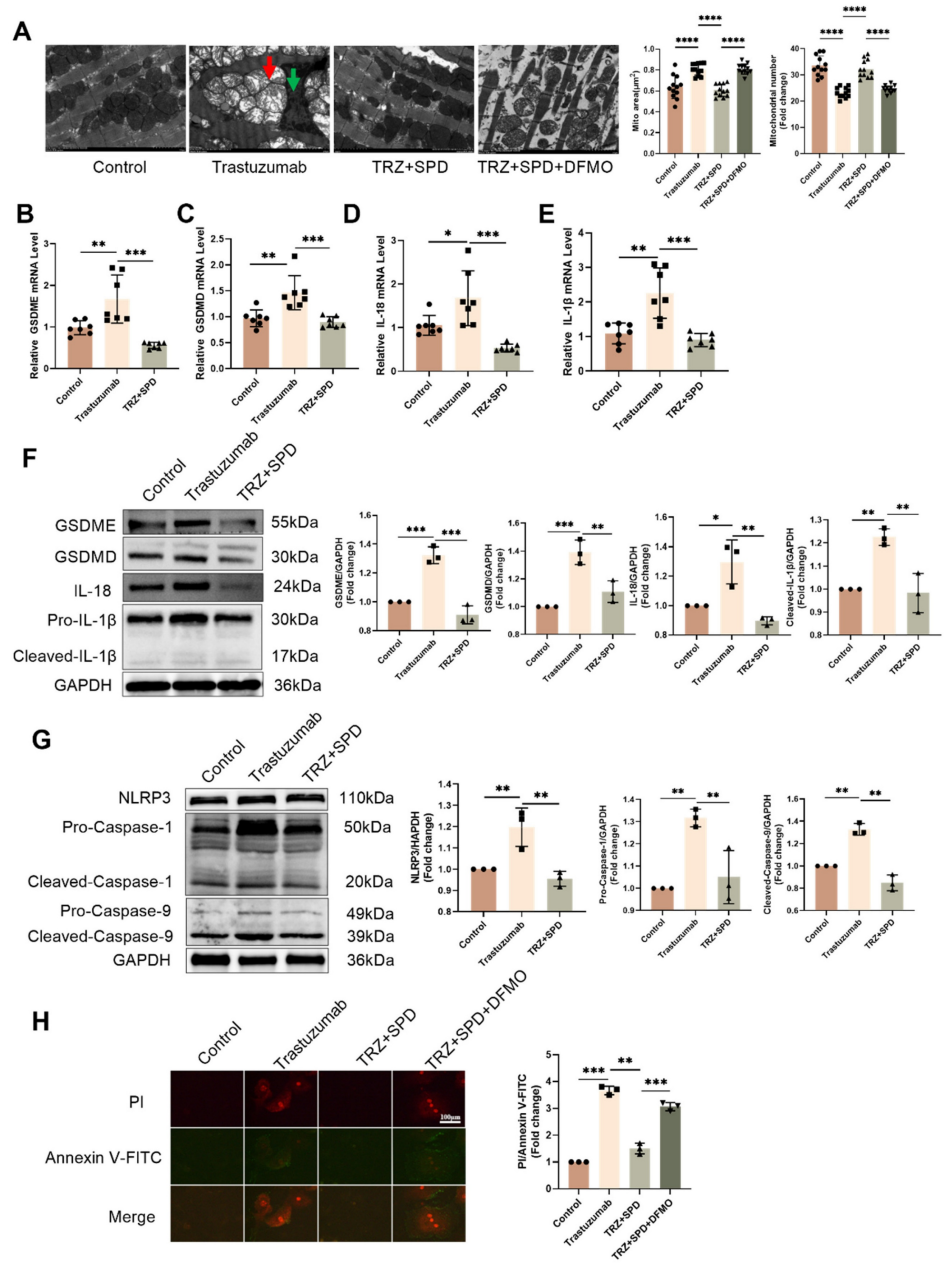
** Exogenous spermidine inhibits trastuzumab-induced inflammation-induced cardiomyocyte pyroptosis.** (A) TEM to observe the cardiac ultrastructure and mitochondrial morphology changes, red arrows represent mitochondrial swelling, green arrows represent nuclear consolidation. Mito area and Mitochondrial number statistics. Mean ± SD (n = 12). **P < 0.01. ANOVA. qPCR was performed to detect the mRNA expression levels of IL-1β (B), GSDMD (C), GSDME (D), and IL-18 (E) in the mice myocardial tissue. Mean ± SD (n=7). *P < 0.05, **P < 0.01 and ***P < 0.001. ANOVA. (F) Western blotting was performed to analyze the protein expression levels of the GSDME, GSDMD, Pro-IL-18, and IL-1β, in the mice myocardial tissues. Mean ± SD (n = 3). *P < 0.05, **P < 0.01 ***P < 0.001. ANOVA. (G) Protein expression levels of NLRP3, Caspase-1, and Cleaved-Caspase-9 were analyzed by western blotting in mice myocardial tissues. Mean ± SD (n = 3). **P < 0.01. *ANOVA*. (H) PI/Annexin V-FITC fluorescence staining to detect changes in cellular pyroptosis in trastuzumab-treated NRCMs, scale bar = 100 µm. Mean ± SD (n = 3). **P < 0.01 and ***P < 0.001. ANOVA.

To further investigate the toxic effects of trastuzumab on the heart, we examined the ultrastructure of cardiomyocytes under transmission electron microscopy. Trastuzumab-administered cardiomyocytes exhibited nuclear consolidation and highly swollen mitochondria, leading to cardiomyocyte death. Obvious contraction bands were visible, indicating cardiac abnormalities. Furthermore, in the TRZ treatment group, the area of myocardial mitochondria increased significantly, but the number decreased significantly. The structure of cardiomyocytes returned to normal after spermidine intervention (Fig. 2A). Subsequently, qPCR revealed that the mRNA expression of GSDMD, GSDME, IL-18, and IL-1β in cardiomyocytes of the trastuzumab-dosed group significantly increased compared with the control group. However, the mRNA expression of GSDMD, GSDME, IL-18, and IL-1β in cardiomyocytes of the trastuzumab group significantly decreased after spermidine intervention compared with the trastuzumab group (Fig. 2B-Fig. 2E). Western blot analysis similarly showed that exogenous spermidine inhibited the trastuzumab-induced increase in expression of pyroptosis pathway proteins, including GSDMD, GSDME, Pro-IL-18, and IL-1β (Fig. 2F). We then examined the expression of inflammatory vesicles NLRP3, Caspase1, and Cleaved-Caspase9 in myocardial tissues of mice. The WB results demonstrated a significant increase in the expression of NLRP3, Pro-Caspase1, and Cleaved-Caspase9 proteins in the myocardial tissues of mice in the trastuzumab group compared with the control group, whereas exogenous spermidine significantly decreased after intervention (Fig. 2G). This suggests that exogenous spermidine significantly inhibits trastuzumab-induced inflammatory responses in cardiomyocytes. Finally, Annexin V-FITC/PI staining of NRVMs showed significantly enhanced red fluorescence and pyroptosis in the trastuzumab group compared with the control group, while spermidine intervention inhibited trastuzumab-mediated cardiomyocyte pyroptosis (Fig. 2H).

### 3.3. Trastuzumab induces mitochondrial damage in mouse myocardium

**Figure 3:**
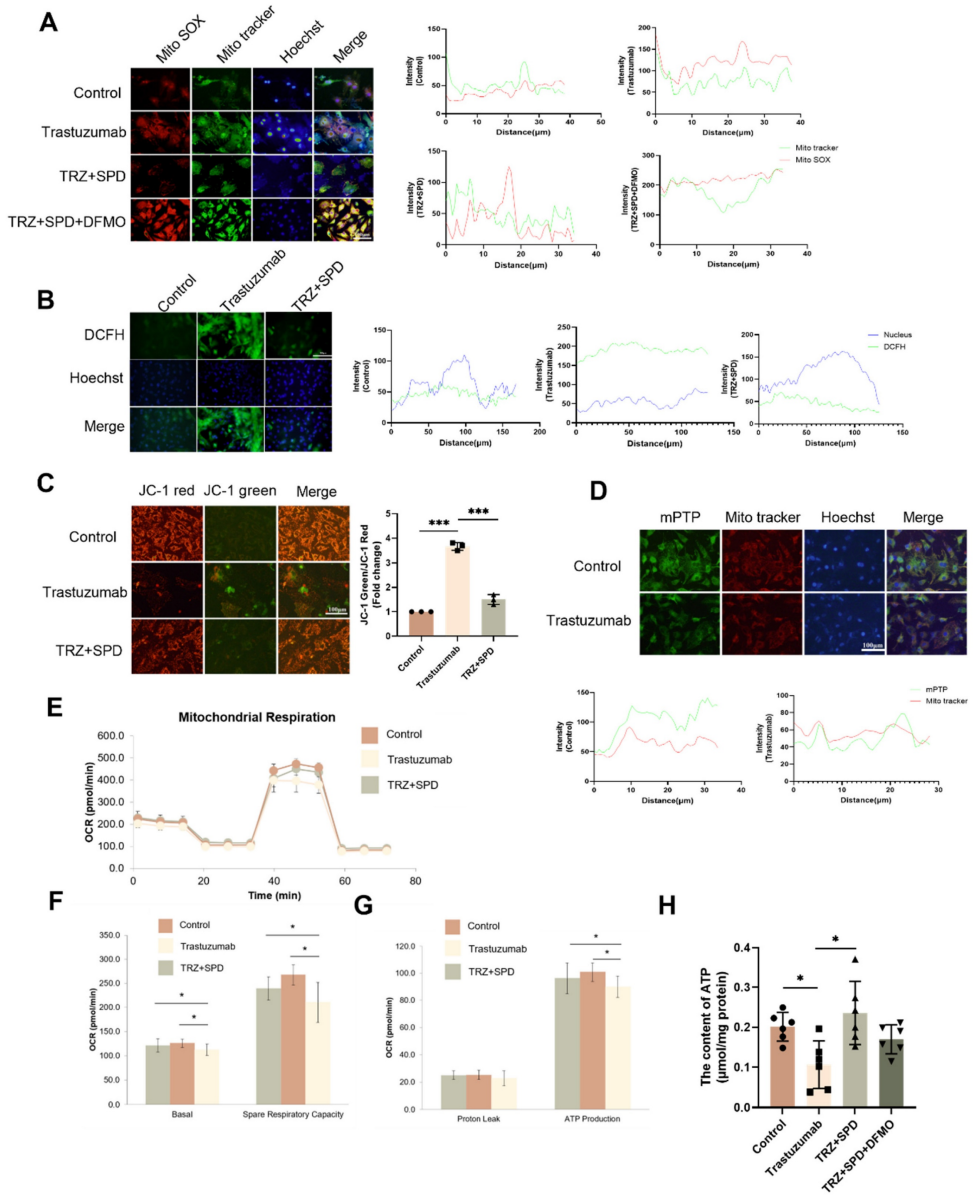
** Effects of trastuzumab on mitochondrial function in mice myocardial tissue.** (A) MtioSOX fluorescent probe to detect mitochondrial ROS production in NRCMs, scale bar = 100 µm. (B) DCFH fluorescent probe to detect ROS levels within NRCMs, scale bar = 500 µm. (C) JC-1 fluorescent probe to detect changes in mitochondrial membrane potential in NRCMs after TRZ treatment, scale bar = 100 µm. Mean ± SD (n=3). ***P < 0.001. ANOVA. (D) mPTP assay kit to detect the opening of the mitochondrial permeability transition pore in NRCMs after TRZ treatment, scale bar = 100 µm. Mitochondrial oxygen consumption rate (OCR) (E), basal respiratory capacity and spare respiratory capacity (F), as well as proton leak and ATP production (G) were measured in 200 µM TRZ-treated HL-1 cells for 48 h by Seahorse. Mean ± SD (n=5), *P < 0.05 and **P < 0.01. ANOVA. (H) ATP assay kits were used to detect ATP levels in the myocardial tissues of mice in each experimental group. Mean ± SD (n=6), *P < 0.05. ANOVA.

During cardiomyocyte pyroptosis, we observed altered myocardial mitochondrial morphology and swelling, prompting an exploration of whether trastuzumab induces mitochondrial damage in cardiomyocytes. Administration of TRZ in NRVMs resulted in an increase in cellular mitochondrial reactive oxygen species production, exogenous spermidine intervention reduced the TRZ-induced enhancement of mitochondrial oxidative stress, and the inhibitor of spermine synthesis, DFMO, abolished the role of SPD (Fig. 3A). Concurrently, TRZ administration in NRVMs led to an increase in cellular reactive oxygen species (Fig. 3B). We further assessed changes in mitochondrial membrane potential using the JC-1 fluorescent probe, revealing increased green fluorescence, decreased red fluorescence, and diminished mitochondrial membrane potential in the TRZ-treated group (Fig. 3C). Using the mPTP fluorescent probe to measure mitochondrial membrane permeability transporter pore opening, we observed weakened green fluorescence in the TRZ-treated group, indicating heightened mitochondrial membrane permeability transporter pore opening and enhanced mitochondrial membrane permeability in the TRZ group (Fig. 3D). It is evident that TRZ induces a decrease in membrane potential and an increase in mitochondrial membrane permeability in NRVMs, ultimately leading to cell death. To further investigate the effect of TRZ on cardiac mitochondrial function, we examined mitochondrial function using Seahorse. The OCR curves indicated that TRZ treatment decreased the basal respiratory rate and maximal respiratory capacity of mitochondria in cardiomyocytes (Fig. 3E). Simultaneously, TRZ significantly reduced mitochondrial reserve respiratory function (Fig. 3F). Additionally, TRZ treatment significantly diminished cardiomyocyte mitochondrial ATP production capacity, with no significant change in proton leakage (Fig. 3G). Furthermore, we examined ATP content in myocardial tissues of mice, revealing a significant reduction in ATP content in TRZ group mice compared to control mice, an effect reversed by exogenous spermidine intervention, while DFMO abolished the effect of SPD (Fig. 3H). These results suggest that TRZ administration impairs the function of myocardial mitochondria and cardiomyocyte mitochondria in mice, causing mitochondrial damage.

### 3.4. Trastuzumab induces mitochondrial quality control changes in mice myocardium

To further investigate the mechanism of myocardial mitochondrial damage induced by trastuzumab, we assessed its impact on mitochondrial quality control. Trastuzumab administration markedly reduced the mRNA expression of proteins associated with mitochondrial biosynthesis, namely PGC-1α, NRF-1, and TFAM, in comparison to the control group, as demonstrated by qPCR assay.

**Figure 4:**
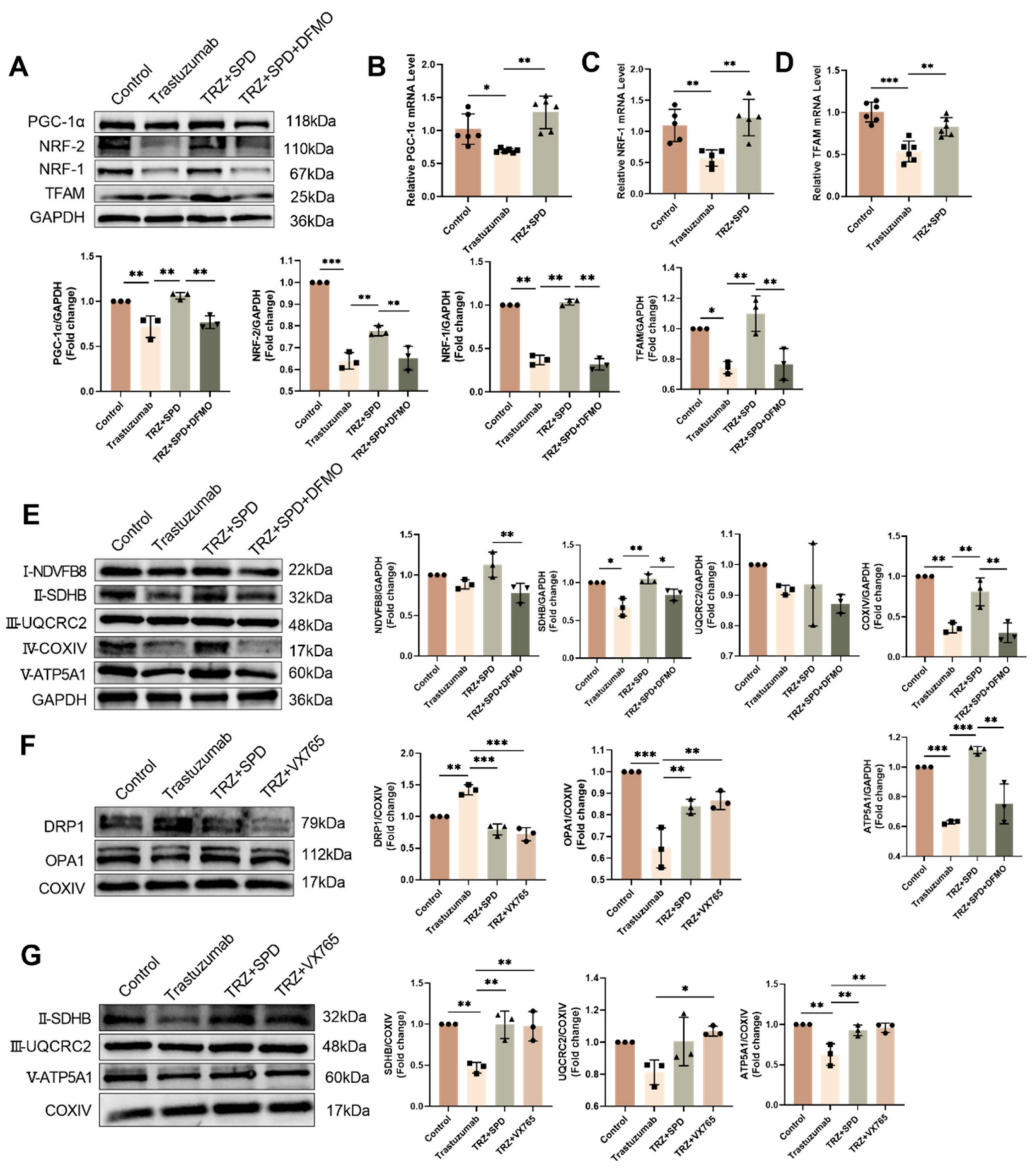
** Effect of trastuzumab on mitochondrial protein expression.** (A) Western blotting analysis of PGC -1α, NRF-1, NRF-2, and TFAM protein expression levels in mice myocardial tissues. Mean ± SD (n = 3). **P < 0.01 and ***P < 0.001. ANOVA. qPCR was performed to detect mRNA levels of PGC-1α (B), NRF-1 (C), and TFAM (D) in mice myocardial tissue. Mean ± SD (n=6). *P < 0.05, **P < 0.01 and ***P < 0.001. ANOVA. (E) Western blotting was performed to analyze the protein expression of I-NDVFB8, II-SDHB, III-UQCRC2, IV-COXIV, and V-ATP5A1 in the mice myocardial tissues. Mean ± SD (n=3). *P < 0.05, **P < 0.01 and ***P < 0.001. ANOVA. (F) Western blotting was performed to analyze the protein expression of DRPA and OPA1 in the HL-1 cells. Mean ± SD (n = 3). ** P < 0.01 and ***P < 0.001. ANOVA. (G) Western blotting to analyze the protein expression levels of II-SDHB, III-UQCRC2, and V-ATP5A1 in HL-1 cells. Mean ± SD (n = 3). *P < 0.05 and **P < 0.01. ANOVA.

Intervention with exogenous spermidine significantly mitigated the effects induced by trastuzumab (Fig. 4A-Fig. 4C). Subsequently, we analyzed the expression of complex proteins involved in the myocardial mitochondrial electron transport chain. Administration of TRZ significantly diminished the protein abundance of complexes I-NDVFB8, II-SDHB, IV-COXIV, and V-ATP5A1. however, the protein abundance of complex III-UQCRC2 remained unchanged. Supplementing trastuzumab administration with exogenous spermidine intervention significantly mitigated the protective effect of TRZ, and the protective effect of spermine was nullified by additional DFMO treatment (Fig. 4D). Western blot results also showed that trastuzumab reduced the protein abundance of PGC-1α, NRF-1, NRF-2, and TFAM in mice myocardial tissues, and that exogenous spermidine treatment significantly inhibited the action of TRZ. Spermidine synthesis inhibitor DFMO treatment abolished the protective effect of spermidine (Fig. 4E). Additionally, both SPD and VX765 markedly mitigated the TRZ-induced increase in DRP1 expression and decrease in OPA1 expression (Fig. 4F). We next extracted mitochondrial proteins from HL-1 cardiomyocytes, and WB results showed that TRZ significantly reduced mitochondrial protein expression of SDHB, ATP5A1, UQCRC2, and TFAM, and the addition of exogenous spermidine and the pyroptosis inhibitor VX765 interventions both abolished the effects of TRZ (Fig. 4G). The findings suggest that TRZ induces a reduction in mitochondrial biosynthesis, a decrease in mitochondrial fusion, an increase in fission, and alterations in mitochondrial dynamics and quality control in cardiomyocytes. Furthermore, exogenous spermidine intervention provides protection against TRZ-induced injury in cardiomyocytes.

### 3.5. Trastuzumab induces cardiac injury in SIRT3 knockout mice

**Figure 5:**
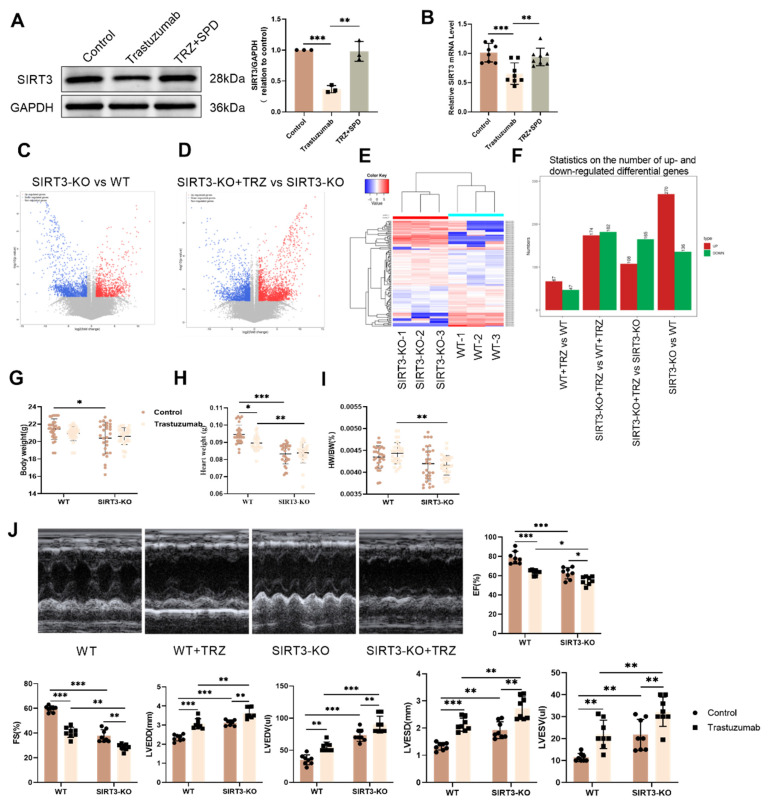
** Trastuzumab induced myocardial injury in SIRT3 knockout mice.** (A) Protein expression levels of SIRT3 in mice myocardial tissues were analyzed by western blotting. Mean ± SD (n = 3). **P < 0.01 and ***P < 0.001. ANOVA. (B) qPCR detection of mRNA expression levels of SIRT3 in mice myocardial tissues. Mean ± SD (n=8). **P < 0.01 and ***P < 0.001. ANOVA. (C) Volcano plots showing differential gene expression in myocardial tissues of SIRT3-KO mice and WT mice. (D) Volcano plots showing differential expression of genes in myocardial tissues of SIRT3-KO mice. (E) Heatmap showing clustered expression of genes in myocardial tissues of SIRT3-KO mice and WT mice. Effects of TRZ treatment on body weight (BW) (F) RNA-seq analysis of gene differential expression in each experimental group. (G) Body weight (BW), (H) heart weight (HW) and (I) heart weight to body weight ratio (HW/BW) in WT and SIRT3-KO mice. Mean ± SD (n=29:29:29:26). *P < 0.05, **P < 0.01 and ***P < 0.001. ANOVA. (J) Mice echocardiography was performed to assess cardiac function in each experimental group as follows: ejection fraction (EF), short-axis shortening (FS), left ventricular end-diastolic internal diameter (LVEDD), left ventricular end-diastolic volume (LVEDV), left ventricular end-systolic internal diameter (LVESD), and left ventricular end-systolic volume (LVESV). Mean ± SD (n=8). **p < 0.01 and ***p < 0.001. ANOVA.

Given the pivotal role of SIRT3 as a regulator within mitochondria, our investigation sought to determine whether TRZ induces mitochondrial damage through modulation of SIRT3 expression. Subsequent to TRZ treatment, both mRNA and protein levels of SIRT3 exhibited a significant reduction in myocardial tissues (Fig. 5A-Fig. 5B). Systemic SIRT3 knockout mice were generated, and RNA-seq sequencing revealed 270 significantly up-regulated genes and 136 significantly down-regulated genes in the SIRT3-KO group compared to the wild type (Fig. 5C-Fig. 5F). Following TRZ administration, 108 genes showed significant up-regulation, and 165 genes exhibited significant down-regulation compared to the SIRT3-KO group (Fig. 5F). Subsequently, We observed a significant reduction in body weight in SIRT3 knockout mice compared with wild-type mice, both in the presence and absence of TRZ treatment (Fig. 5G). Following TRZ administration, the heart size was significantly smaller in the SIRT3 knockout group compared with the wild-type mice TRZ group (Fig. 5H). Additionally, the addition of TRZ treatment to SIRT3 knockout mice resulted in a notable decrease in the heart weight to body weight ratio in both groups compared with the WT mice TRZ group (Fig. 5I). Cardiac ultrasound results indicated a slight reduction in cardiac function in SIRT3 knockout mice compared with wild-type mice. However, TRZ treatment significantly further diminished cardiac function in comparison with both the SIRT3-KO mice control and WT mice TRZ groups. This was evidenced by significant reductions in ejection fraction (EF) and short-axis shortening (FS) and significant increases in LVEDD, LVESD, LVEDV, and LVESV (Fig. 5J). The results indicated that SIRT3 knockout had a minimal effect on growth and development and cardiac function. However, the damage was exacerbated by the addition of TRZ treatment, rendering the SIRT3 knockout mice more vulnerable to adverse factors. The foregoing results suggest that TRZ treatment leads to a decrease in SIRT3 expression in the heart, and that SIRT3 knockout exacerbates cardiac injury in the presence of TRZ administration.

### 3.6. Protective effect of SIRT3 on trastuzumab-induced myocardial pyroptosis

**Figure 6:**
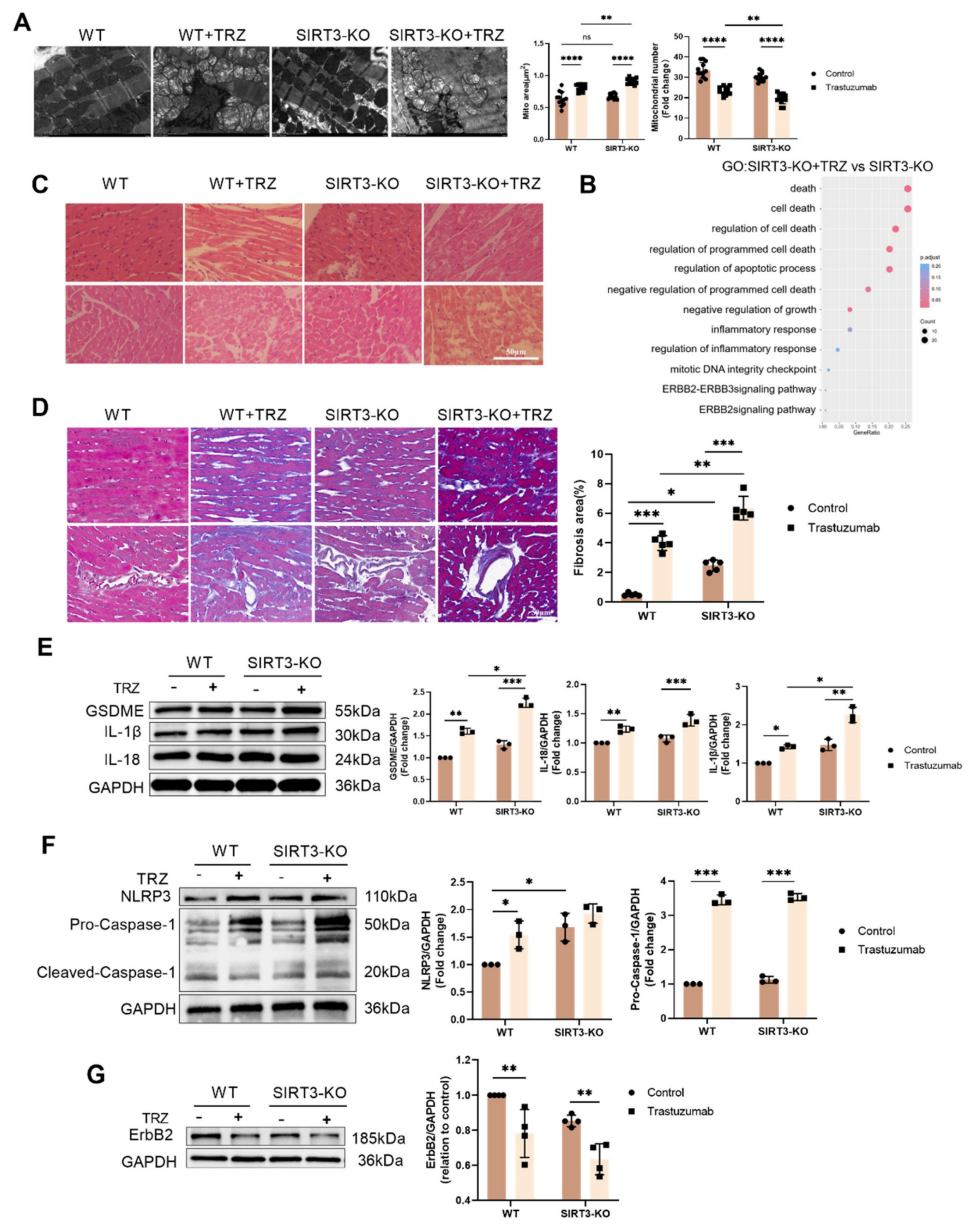
** Trastuzumab induces myocardial pyroptosis in SIRT3-KO mice.** (A) Transmission electron microscopy observation of cardiac ultrastructure and mitochondrial morphological changes. Mito area and Mitochondrial number statistics. Mean ± SD (n = 12). **P < 0.01. ANOVA. (B) Results of gene function differences between TRZ-treated and control groups in SIRT3-KO mice analyzed by GO enrichment. (C) Representative HE staining of ventricular slices of each group (n=5), scale bar = 50 µm. (D) Representative Masson staining of ventricular slices of each group (n=5), scale bar = 50 µm. Mean ± SD. *P < 0.05, **P < 0.01 and ***P < 0.001. ANOVA. (E) Protein expression levels of pyroptosis pathway proteins GSDME, Pro-IL-18, and Pro-IL-1β were analyzed by western blotting in myocardial tissues. Mean ± SD (n = 3). *P < 0.05, **P < 0.01 and ***P < 0.001. ANOVA. (F) Protein expression levels of NLRP3 and Pro-Caspase-1 in myocardial tissues of each SIRT3-KO mouse and wild-type mouse were analyzed by western blotting. Mean ± SD (n = 3). *P < 0.05 and ***P < 0.001. ANOVA. (G) Protein expression levels of ErbB2 in myocardial tissues of each SIRT3-KO mice and wild-type mice were analyzed by western blotting. Mean ± SD (n = 3). **P < 0.01. ANOVA.

To explore the protective effect of SIRT3 against TRZ-induced myocardial injury, we examined the ultrastructure of cardiomyocytes using transmission electron microscopy. We observed that, in comparison to wild-type mice, cardiac myofibrils in SIRT3 knockout mice exhibited orderly alignment and normal mitochondrial morphology with no discernible changes. However, after TRZ administration and treatment, evident mitochondrial swelling between myofilaments and nuclear consolidation of cardiomyocytes were observed, indicating the occurrence of myocardial pyroptosis (Fig. 6A). Cardiac H&E staining showed that the myocardial structure of mice in the trastuzumab group was sparse and disorganized, whereas myofilaments were neatly arranged and the cytoplasmic staining was homogeneous in the SIRT3-KO group, but sparse and disorganized in the SIRT3-KO+TRZ group (Fig. 6C).

Meanwhile, Masson staining results showed that myocardial tissue collagen deposition was increased in the SIRT3-KO group of mice, and the level of fibrosis was aggravated in both the interstitium and the perivascular area, compared with the control group of WT mice. In addition, myocardial tissue collagen deposition was increased in SIRT3-KO+TRZ group mice, both interstitial and perivascular, and fibrosis levels were aggravated, both when compared with WT+TRZ group mice and when compared with SIRT3-KO group mice (Fig. 6D). The RNA-seq results were subjected to GO enrichment analysis in the TRZ-treated group of SIRT3-KO mice versus the SIRT3-KO group. The analysis revealed a close association with processes related to cell death, the ErbB2-ErbB3 signaling pathway, and the inflammatory response (Fig. 6B). Subsequent examination of pyroptosis pathway protein expression in myocardial tissues revealed that in SIRT3-KO mice, TRZ administration significantly increased the protein abundance of GSDME, Pro-IL-18, and Pro-IL-1β. Moreover, protein expression was also significantly elevated in the TRZ group compared with the WT mice (Fig. 6E). In both wild-type and SIRT3-KO mice, TRZ administration significantly increased the expression of NLRP3 inflammatory vesicles and Pro-Caspase-1 (Fig. 6F). Furthermore, in both wild-type and SIRT3-KO mice, TRZ administration significantly decreased the expression of ErbB2 protein (Fig. 6G). The heightened cardiac inflammatory response and pyroptosis observed in mice following TRZ administration treatment, along with the exacerbated injury in SIRT3-KO mice, underscore the crucial protective role of SIRT3 against trastuzumab-induced myocardial pyroptosis.

### 3.7. Mitochondrial protection by the AMPK-PGC-1a-Sirt3 pathway in TRZ-treated cells

**Figure 7:**
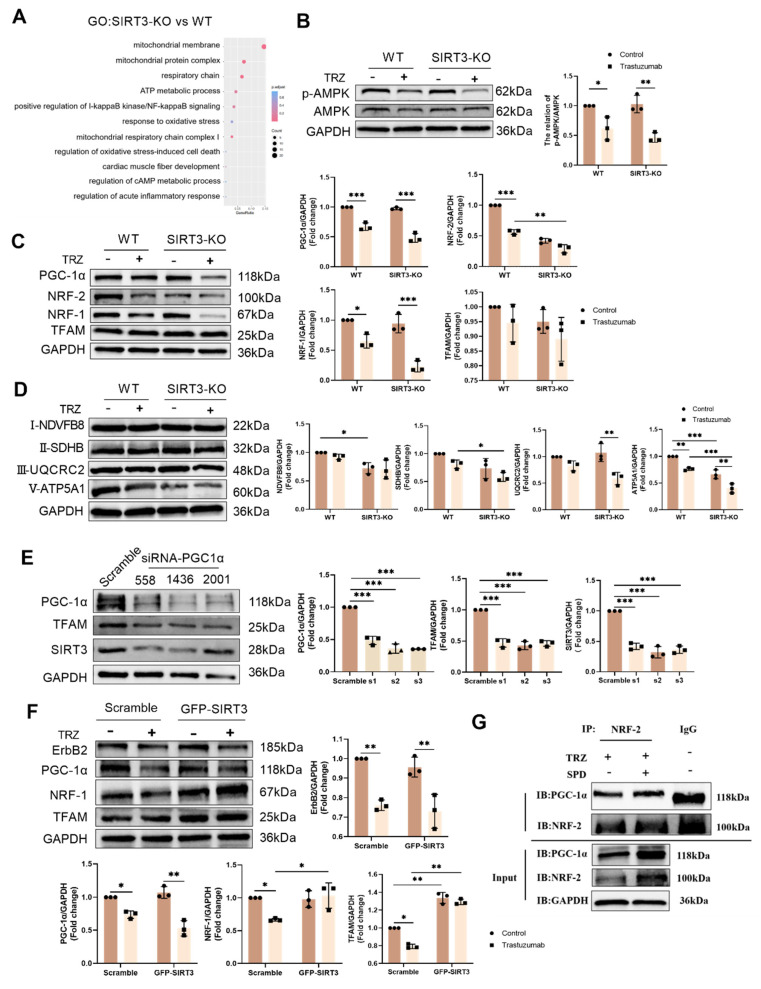
** Trastuzumab induces reduced mitochondrial biosynthesis through activation of AMPK phosphorylation and reduced mitochondrial electron transport chain complex proteins in which SIRT3 plays a protective role.** (A) Gene function in SIRT3-KO mice and WT mice enriched by GO analysis. (B) Protein phosphorylation levels of AMPK in myocardial tissues of SIRT3-KO mice and wild-type mice were analyzed by western blotting. Mean ± SD (n = 3). *P < 0.05 and **P < 0.01. ANOVA. (C) Protein expression levels of mitochondrial biosynthetic pathway proteins PGC-1α, NRF-2, NRF-1, and TFAM were analyzed by western blotting in myocardial tissues of SIRT3-KO mice and wild-type mice. Mean ± SD (n = 3). *P < 0.05, **P < 0.01 and ***P < 0.001. ANOVA. (D) Western blotting analysis of mitochondrial complex proteins I-NDVFB8, II-SDHB, III-UQCRC2, and V-ATP5A1 protein expression levels in myocardial tissues of SIRT3-KO mice and wild-type mice. Mean ± SD (n = 3). *P< 0.05, **P < 0.01 and ***P < 0.001. ANOVA. (E) Protein expression levels of the mitochondrial biosynthesis pathway proteins PGC-1α, SIRT3, and TFAM were analyzed by western blotting in HL-1 cardiomyocytes after the knockdown of PGC-1α using siRNA. Mean ± SD (n = 3). ***P < 0.001. ANOVA. (F) western blotting in HL-1 cardiomyocytes after adenoviral infection with SIRT3 overexpression was used to analyze the levels of HL-1 cellular ErbB2, PGC-1α, NRF-1, and TFAM protein expression levels. Mean ± SD (n = 3). *P< 0.05 and **P < 0.01. ANOVA. (G) Interaction of PGC-1α and NRF-2 after exogenous spermidine intervention in HL-1 cardiomyocytes was examined by co-IP.

Illustrated in Figure 7, our results depict the GO enrichment analysis of gene function disparities between SIRT3-KO mice and WT mice. The enrichment was observed in mitochondrial respiratory chain complex 2 biogenesis, positive regulation of oxidoreductase activity, and cAMP response (Fig. 7A). Subsequent investigations revealed that trastuzumab significantly decreased AMPK phosphorylation, and SIRT3 knockdown had no impact on AMPK phosphorylation (Fig. 7B). AMPK phosphorylation was enhanced after administration of the AMPK agonist AICAR 100 µM treatment in NRCMs (Supplemental Fig. 1A). TRZ-induced reduction of ErbB2 expression could not be reversed by AICAR, but AICAR treatment inhibited TRZ-mediated reduction of ULK1 and LC3II/I (Supplemental Fig. 1B). Concurrently, AICAR treatment facilitated the recovery of TRZ-injured mitochondrial membrane potential (Supplemental Fig. 1C) and mitigated mitochondrial oxidative stress (Supplemental Fig. 1D). In WT mice, TRZ decreased the expression of PGC-1α, NRF-1, and NRF-2. SIRT3 knockout mice exhibited no significant changes compared to WT mice; however, TRZ administration to SIRT3 knockout mice resulted in a significant decrease in the expression of PGC-1α, NRF-1, and NRF-2. Notably, PGC-1α and NRF-1 expression were significantly reduced compared to the WT+TRZ group (Fig. 7C, Supplemental Fig. 1E). Additionally, we assessed the expression of mitochondrial respiratory chain complex proteins and observed a significant reduction in the expression of complexes II-SDHB, III-UQCRC2, and V-ATP5A1 after TRZ treatment in SIRT3 knockout mice. Conversely, no significant change was observed for complex I-NDVFB8 (Fig. 7D). In HL-1 cardiomyocytes, siRNA-mediated knockdown of PGC-1α significantly downregulated the expression of TFAM and SIRT3 in the cells (Fig. 7E). Furthermore, adenoviral overexpression of SIRT3 in HL-1 cardiomyocytes infected with SIRT3 did not mitigate TRZ-induced reductions in the expression of ErbB2 and PGC-1α. However, it significantly increased TRZ-mediated reductions in the expression of NRF-1 and TFAM proteins. This suggests that SIRT3 may function as a downstream factor of ErbB2 and PGC-1α (Supplemental Fig. 1F, Fig. 7F). To further explore the mechanism of action of exogenous spermidine in modulating myocardial injury by trastuzumab, we examined the interaction between PGC-1α and NRF-2 after exogenous spermidine treatment in HL-1 cells, and the results showed that the interaction between PGC-1α and NRF-2 was enhanced by the addition of exogenous spermidine intervention compared with that in the trastuzumab group (Fig. 7G). The aforementioned findings indicate that trastuzumab inhibits the AMPK pathway, leading to reduced mitochondrial biosynthesis and disruptions in mitochondrial electron transport chain complexes. Conversely, activation of the AMPK pathway appears to play a protective role.

### 3.8. SIRT3 plays a protective role in TRZ-mediated oxidative stress

**Figure 8:**
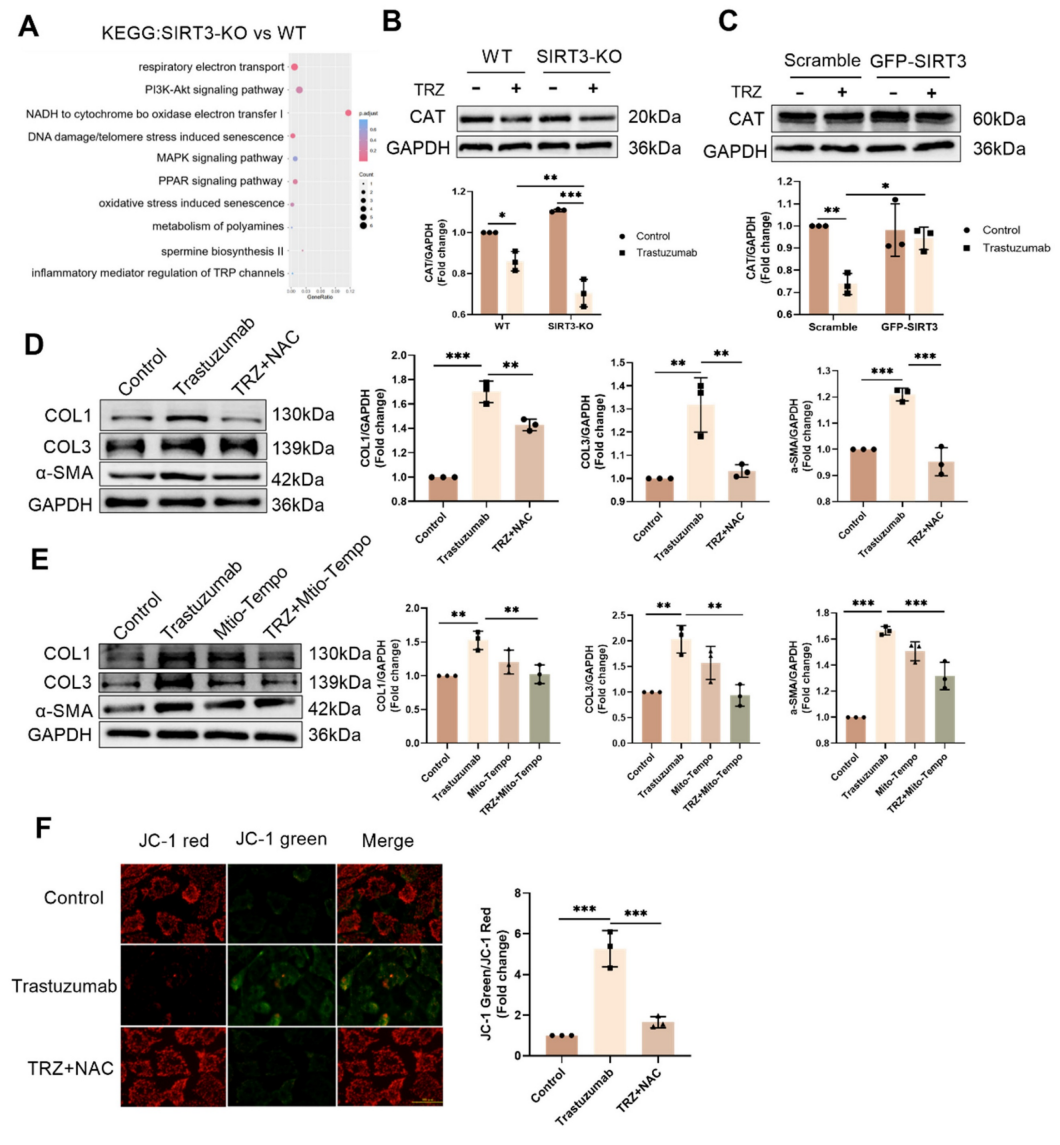
** SIRT3 plays a protective role in trastuzumab-mediated oxidative stress in cardiomyocytes and scavenging of both cytosolic and mitochondrial reactive oxygen species inhibits TRZ-mediated fibrosis in primary fibroblasts.** (A) Differences in gene function in WT mice and SIRT3-KO mice were analyzed by KEGG enrichment. (B) Protein expression levels of CAT in myocardial tissues of SIRT3-KO mice and wild-type mice were analyzed by western blotting. Mean ± SD (n = 3). *P < 0.05, **P < 0.01 and ***P < 0.001. ANOVA. (C) Protein expression levels of COL1, COL3, and α-SMA were analyzed by western blotting in primary fibroblasts treated with TRZ and the cellular reactive oxygen scavenger NAC. Mean ± SD (n = 3). *P < 0.05, **P < 0.01 and ***P < 0.001. ANOVA. (D) Protein expression levels of CAT were analyzed by western blotting after infection of adenovirus overexpressing SIRT3 in HL-1 cardiomyocytes. Mean ± SD (n = 3). *P < 0.05 and **P < 0.01. ANOVA. (E) Protein expression levels of COL1, COL3, and α-SMA were analyzed by western blotting in primary fibroblasts after treatment with TRZ and the Mito-Tempo, a mitochondrial reactive oxygen scavenger. Mean ± SD (n = 3). **P < 0.01 and ***P < 0.001. ANOVA. (F) JC-1 Activity Assay Kit for detecting mitochondrial membrane potential changes in primary fibroblasts, scale bar = 100 µm. Mean ± SD (n = 3). ***P < 0.001. ANOVA.

To delve deeper into the mechanism of TRZ-induced myocardial mitochondrial damage, we extended our exploration to primary fibroblasts. Analyzing SIRT3-KO mice and WT mice through KEGG enrichment revealed a significant correlation with oxidative stress (Fig. 8A). In WT mice, TRZ administration led to a significant reduction in the protein abundance of CAT.

While there was no significant change in the protein expression of CAT in SIRT-KO mice compared with WT mice, CAT expression was significantly reduced by TRZ treatment. Additionally, CAT protein expression was significantly decreased in the TRZ group of SIRT3-KO mice compared with the TRZ group of WT mice (Fig. 8B). In primary fibroblasts, the introduction of the cellular reactive oxygen scavenger NAC treatment markedly inhibited TRZ-induced upregulation of COL1, COL3, and α-SMA expression (Fig. 8C). In HL-1 cells, the overexpression of SIRT3 hindered the TRZ-mediated reduction of CAT expression (Fig. 8D). In primary fibroblasts, the inclusion of the mitochondrial scavenger Mito-Tempo yielded consistent results with the addition of NAC, both of which inhibited trastuzumab-mediated enhancement of fibrosis in fibroblasts (Fig. 8E). Additionally, NAC treatment exacerbated the TRZ-mediated decrease in fibroblast membrane potential (Fig. 8F). The aforementioned results indicate that SIRT3 functions protectively in TRZ-induced oxidative stress, and the inhibition of oxidative stress mitigates TRZ-induced enhancement of fibrosis in fibroblasts.

### 3.9. TRZ promotes mitochondrial division through the PI3K-AKT pathway and thus leads to enhanced cellular fibrosis

**Figure 9:**
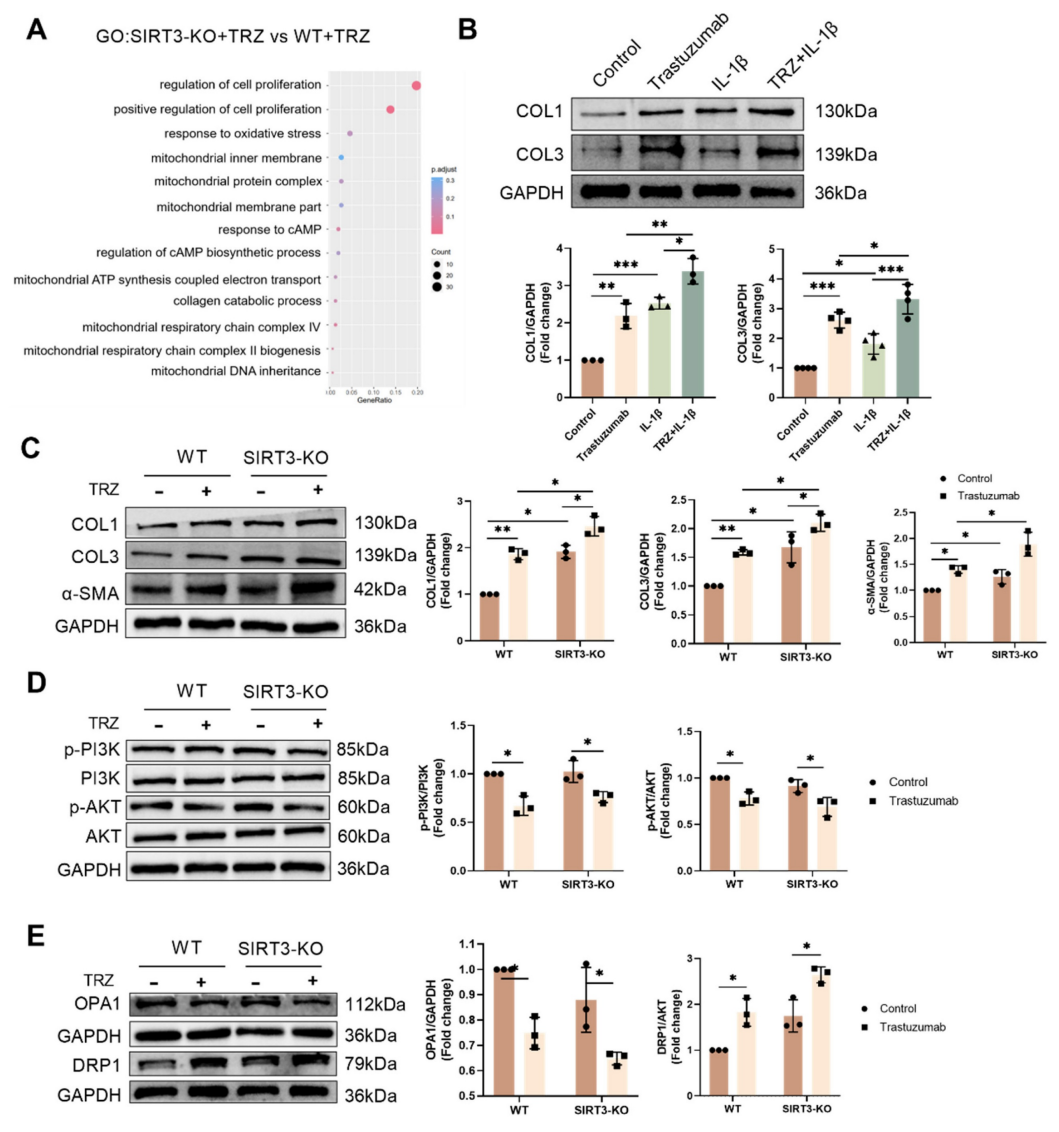
** Trastuzumab induces fibrosis in primary mammary mice fibroblasts by inhibiting the PI3K-AKT pathway.** (A) Differences in gene function between the two groups of mice after TRZ treatment were analyzed by GO enrichment in WT mice and SIRT3-KO mice. (B) Protein expression levels of fibrotic proteins COL1 and COL3 were analyzed by western blotting in primary fibroblasts after induction with the addition of the inflammatory factor IL-1β. Mean ± SD (n = 3). *P < 0.05, **P < 0.01 and ***P < 0.001. ANOVA. (C) Protein expression levels of fibrotic proteins COL1, COL3, and α-SMA were analyzed by western blotting after TRZ treatment in WT and SIRT3-KO mice. Mean ± SD (n = 3). *P < 0.05 and **P < 0.001. ANOVA. (D) Phosphorylation levels of PI3K and AKT were analyzed by western blotting after TRZ treatment in WT and SIRT3-KO mice. Mean ± SD (n = 3). *P < 0.05. ANOVA. (E) Phosphorylation levels of OPA1 and DRP1 were analyzed by western blotting after TRZ treatment in WT and SIRT3-KO mice. Mean ± SD (n = 3). *P < 0.05. ANOVA.

Analyzing the RNA-seq results of the SIRT3-KO+TRZ group versus the WT+TRZ group through GO enrichment, we identified enrichment in the mitochondrial inner membrane and collagen catabolism (Fig. 9A). In fibroblasts, stimulation with the inflammatory factor IL-1β led to increased collagen production and elevated COL1 and COL3 protein expression. Simultaneous treatment with TRZ and IL-1β resulted in significantly higher expression of both COL1 and COL3 compared to treatment with TRZ or IL-1β alone (Fig. 9B). Subsequently, we examined fibrosis in SIRT3-KO mice and observed no significant change in fibrosis after SIRT3 knockout compared to WT mice. However, there was a significant increase in COL1, COL3, and α-SMA expression, along with enhanced fibrosis after TRZ stimulation (Fig. 9C). Existing evidence indicates that the PI3K/AKT pathway is maximally and directly involved in the formation of lung fibrosis or collaborates with other pathways to promote fibrosis development. Consequently, we examined changes in the PI3K/AKT pathway in myocardial tissue and demonstrated that TRZ treatment inhibited PI3K/AKT pathway phosphorylation in both WT mice and SIRT3-KO mice (Fig. 9D). Subsequently, we investigated mitochondrial dynamics and revealed that TRZ mediated a decrease in mitochondrial OPA1 expression, an increase in DRP1 expression, and an increase in mitochondrial fusion-reduced fragmentation in mice myocardium. In SIRT3-KO mice, there were no significant changes in mitochondrial fusion division, and the addition of TRZ treatment resulted in a significant decrease in OPA1 expression and a significant increase in DRP1 expression. Notably, TRZ-treated DRP1 expression was significantly elevated in SIRT3-KO mice compared with the TRZ group of WT mice (Fig. 9E). In fibroblasts, the inclusion of the mitochondrial division inhibitor Mdivi-1 significantly reduced cellular COL1, COL3, and α-SMA expression. Furthermore, it inhibited TRZ-mediated enhancement of COL1, COL3, and α-SMA expression and attenuated TRZ-mediated enhancement of fibrosis in fibroblasts (Supplemental Fig. 2A-Fig. 2D). Additionally, the inclusion of Mdivi-1 treatment inhibited TRZ-mediated enhancement of fibroblast oxidative stress (Supplemental Fig. 2E). In conclusion, trastuzumab inhibits PI3K/AKT pathway phosphorylation, disrupts mitochondrial dynamics, inhibits mitochondrial fusion, promotes mitochondrial fission, and consequently, results in enhanced fibrosis. Inhibition of mitochondrial division mitigates TRZ-mediated enhancement of fibrosis.

### 3.10. Exogenous spermidine protects TRZ-induced myocardial injury via SIRT3

**Figure 10:**
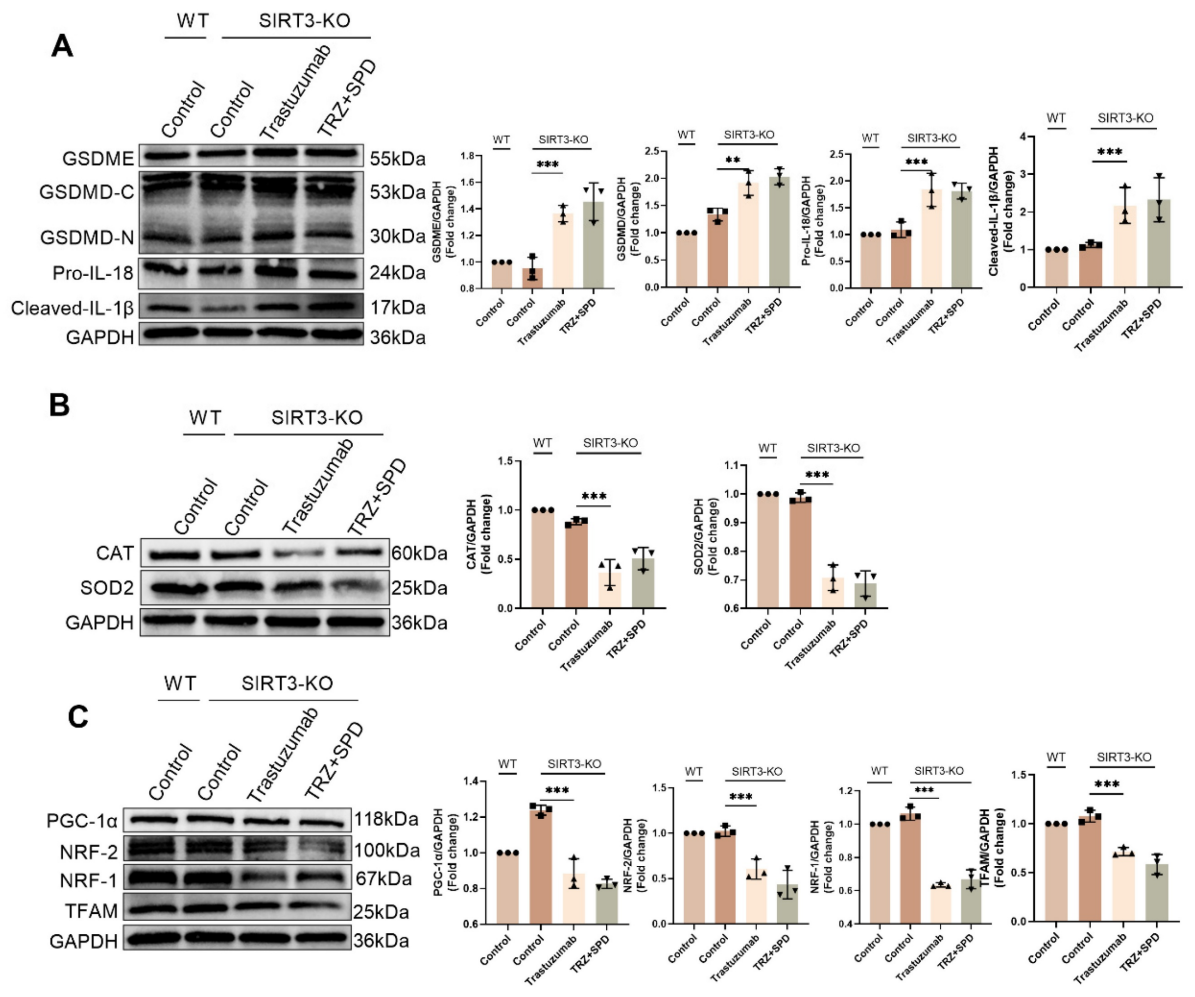
** Exogenous spermidine protects myocardial tissue from trastuzumab injury via SIRT3** (A) Western blotting analysis the protein expression levels of pyroptosis pathway proteins GSDME, GSDMD, Cleaved-IL-1β and Pro-IL-18 in myocardial tissues of WT and SIRT3-KO mice treated with trastuzumab and exogenous spermidine. Mean ± SD (n = 3). **P < 0.01 and ***P < 0.001. ANOVA. (B) Protein expression levels of CAT and SOD2 were analyzed by western blotting in myocardial tissues of WT and SIRT3-KO mice. Mean ± SD (n = 3). ***P < 0.001. ANOVA. (C) Protein expression levels of mitochondrial biosynthesis pathway proteins PGC-1α, NRF-2, NRF-1 and TFAM were analyzed by western blotting in myocardial tissues of WT and SIRT3-KO mice. Mean ± SD (n = 3). ***P < 0.001. ANOVA.

We further explored whether SIRT3 is an important factor in the protection of TRZ-induced myocardial toxicity by exogenous spermidine after adding exogenous spermidine intervention in an injury model of TRZ-induced cardiotoxicity in SIRT3-KO mice. The expression of pyroptosis pathway proteins in mouse myocardial tissues was examined by WB, and the results showed that there was no significant change in the expression of GSDME, GSDMD, Cleaved-IL-1β and Pro-IL-18 in myocardial tissues of the control group of SIRT3-KO mice compared with that of the control group of wild-type mice. In contrast, in SIRT3-KO mice, the protein abundance of GSDME, GSDMD, Cleaved-IL-1β and Pro-IL-18 in myocardial tissues of trastuzumab-treated group was significantly elevated compared to the control group, and there was no significant change in the expression of pyroptosis pathway proteins after the addition of exogenous spermidine intervention (Fig. 10A). In addition, we examined the expression of CAT and SOD2 proteins to detect oxidative stress in mice. The results showed that there were no significant changes in the expression of CAT and SOD2 in myocardial tissues of SIRT3-KO mouse control group compared to wild-type mouse control group. While in SIRT3-KO mice, the protein abundance of CAT and SOD2 in myocardial tissues of trastuzumab-treated group was significantly reduced compared to the control group, and the addition of exogenous spermidine intervention did not result in any significant changes in the protein expression of CAT and SOD2 (Fig. 10B). Finally, we observed the mitochondrial biosynthesis in mouse myocardial tissues, and the results showed that there was no significant change in the expression of PGC-1α, NRF-2, NRF-1 and TFAM in the myocardial tissues of the control group of SIRT3-KO mice, as compared to the control group of wild-type mice. In contrast, in SIRT3-KO mice, the protein abundance of PGC-1α, NRF-2, NRF-1 and TFAM in myocardial tissues of trastuzumab-treated group was significantly reduced compared with the control group, and there was no significant change in the protein expression of PGC-1α, NRF-2, NRF-1 and TFAM after the addition of exogenous spermidine intervention (Fig. 10C). The above results indicated that the effect of exogenous spermidine was attenuated or even disappeared in SIRT3-KO mice, and it was evident that exogenous spermidine was enhancing mitochondrial biosynthesis and ameliorating TRZ-induced oxidative stress and cardiomyocyte pyroptosis through SIRT3.

## 4. Discussion

**Figure 11:**
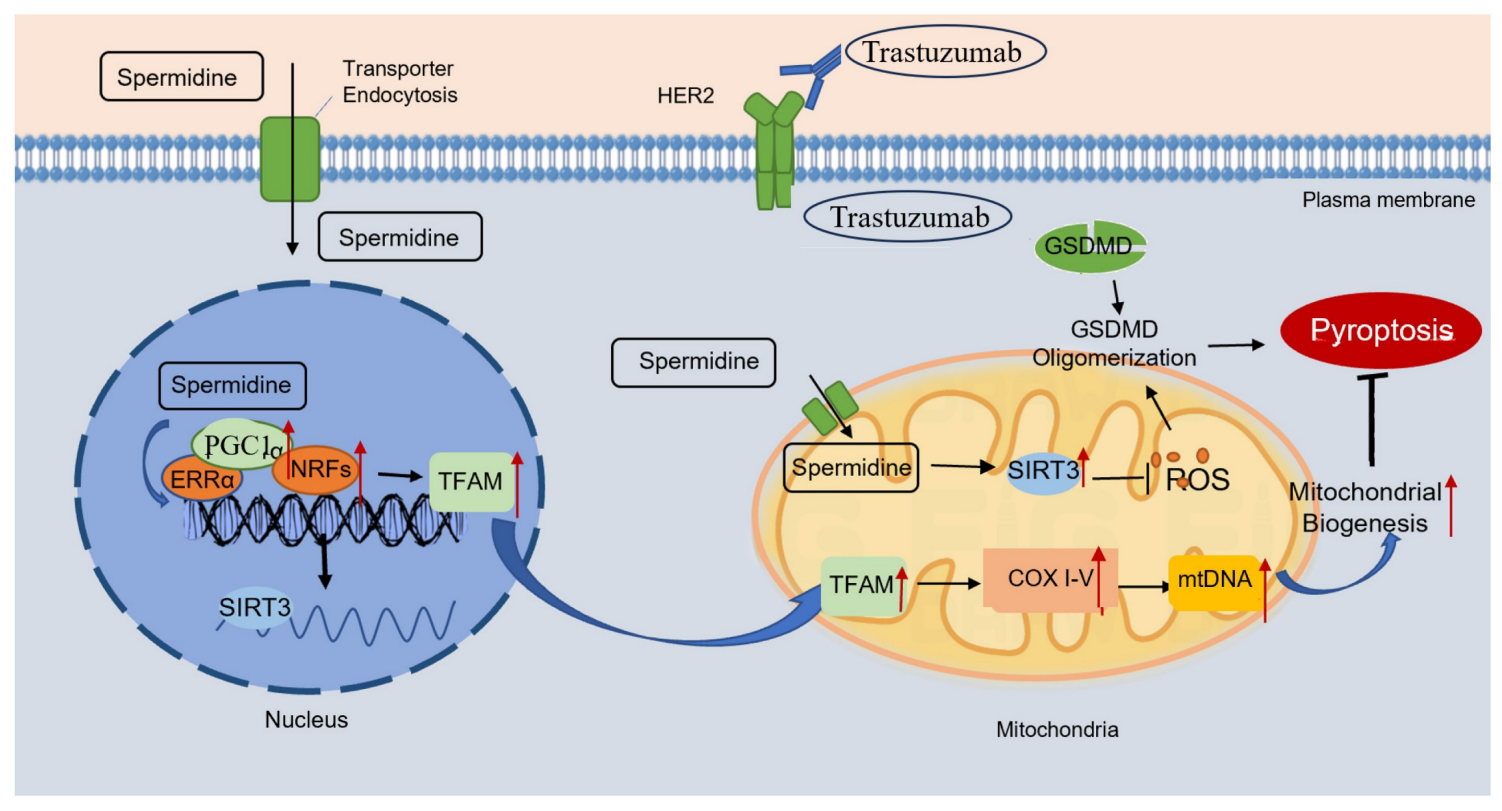
** Exogenous spermidine inhibits trastuzumab-induced cardiomyocyte pyroptosis** Schematic illustration of trastuzumab-induced cardiomyocyte pyroptosis, and exogenous spermidine blocking the action of trastuzumab by promoting mitochondrial biosynthesis. Trastuzumab binds to the HER2 receptor on the cell membrane and induces cleavage of the GSDMD, which in turn causes cardiomyocyte pyroptosis. Exogenous spermidine promotes the expression of PGC-1α in the nucleus, promotes the expression of mitochondrial biosynthesis pathway proteins, inhibits mitochondrial oxidative stress, promotes the function of mitochondria, and inhibits cellular pyroptosis, in which SIRT3 plays an important role. PGC-1α: peroxisome proliIerators-activated receptor γ coactivator lα; ERRα: estrogen receptor related receptor α; NRFs: nuclar respiratory factors; TFAM: mitochondrial transcription factor A; COXI-V: cytochrome c oxidase subunit I-V; GSDMD: gasdermin D; HER2: human epidermal growth factor receptor 2.

TRZ-induced cardiac dysfunction manifests a distinctive clinical phenotype of cardiotoxicity. The cessation of TRZ restores HER2 survival signaling and reverses the decline in cardiac function 36. However, recent retrospective studies caution against assuming that TRZ-induced cardiotoxic effects are fully reversible after treatment termination, as these effects persist and may not return to the normal range, contrary to initial proposals 37, 38. In this study, we demonstrated that TRZ induces cardiomyocyte pyroptosis and explored the cardioprotective effects of exogenous spermidine. The details are as follows: 1. TRZ induced cardiac mitochondrial dysfunction, decreased biosynthesis, enhanced oxidative stress, and cellular death, resulting in left ventricular dysfunction; 2. SIRT3 played a protective role in TRZ-induced cardiotoxicity; 3. Exogenous spermidine improved mitochondrial function, promoted mitochondrial biosynthesis, attenuated oxidative stress, and mitigated cellular pyroptosis by activating the PGC-1α/SIRT3 pathway, effectively preventing TRZ-induced cardiotoxicity (Fig. 11).

While the reversible blockade of ErbB2 by TRZ is widely recognized as synonymous with TIC progression, cardiomyocyte death emerges as an additional contributing factor to cardiotoxicity 39. Mitochondria are crucial organelles for energy production within the cell, contributing to cardiomyocyte death 40. Cellular surveillance of mitochondrial integrity serves as a pivotal checkpoint in the regulation of programmed cell death and the release of inflammatory mediators. Altered mitochondrial homeostasis precipitates a paradigm shift in cell death 22. Notably, this includes the mtROS-dependent N-GSDMD switching between the plasma membrane-associated pyroptosis executioner and the mitochondria-associated initiator of necrotic apoptosis. Excessive ROS, generated by GSDMD through overuse of the electron transport chain, are recruited into mitochondria, subsequently causing GSDMD-mediated cellular pyroptosis 41. In our study, TRZ induced toxic effects in the hearts of C57BL/6J mice, resulting in decreased left ventricular systolic function, enhanced fibrosis, an increased inflammatory response, and cardiomyocyte pyroptosis.

Mitochondrial biosynthesis is crucial for maintaining mitochondrial integrity 21. Current knowledge recognizes that peroxisome proliferator-activated receptor γ coactivator1α (PGC-1α) plays a central role in governing mitochondrial biogenesis and maturation 42. PGC-1α translocates to the nucleus, activating the nuclear transcription factor NRF. This activation promotes the transcription of nuclear-encoded mitochondrial proteins, including those involved in TAC, OXPHOS proteins, mitochondrial antioxidant enzymes, and mitochondrial outer and inner membrane proteins. Simultaneously, mitochondrial transcription factor A (TFAM) is induced, facilitating its binding to mtDNA and driving the replication and transcription of mtDNA. Moreover, PGC-1α synergistically activates ERRa to stimulate the expression of mt SIRT3. This intricate process induces the expression of mitochondrial respiration-associated proteins, promoting mitochondrial biosynthesis through the collaborative action of nuclear DNA (nDNA) and mitochondrial DNA (mtDNA) 43. In our study, we initially discovered that TRZ induces pyroptosis in the mouse heart, resulting in impaired cardiac function and left ventricular systolic dysfunction. Additionally, TRZ treatment leads to a substantial decrease in mRNA and protein expression of PGC-1α, NRF-1, NRF-2, and TFAM in the mouse heart, resulting in diminished mitochondrial biogenesis. This induction of mitochondrial dysfunction in cardiomyocytes is characterized by reduced ATP production and an increase in reactive oxygen species production. Hence, our focus was on enhancing mitochondrial function and mitigating oxidative stress through the augmentation of mitochondrial biogenesis. This approach, in turn, alleviates TRZ-induced myocardial scorch death and cardiac injury.

Mitochondrial biogenesis has been recognized as a factor that may contribute to slowing the aging process 44. The primary objective of mitochondrial biogenesis is to generate new mitochondria through mitochondrial division, thereby sustaining the mitochondrial cycle in collaboration with mitochondrial autophagy 45. Impaired or defective mitochondrial biogenesis is linked to mitochondrial aging or the accumulation of dysfunctional mitochondria characterized by reduced mitochondrial potential or elevated ROS 46. Consequently, these damaged mitochondria disrupt cardiomyocyte metabolism, leading to mitochondrial apoptosis or necrosis through mPTP opening 47. Therefore, the activation of mitochondrial biogenesis serves as a protective mechanism to diminish the vulnerability of cardiomyocytes to stress. For instance, enhanced mitochondrial biogenesis shields cardiomyocytes from ischemia-reperfusion injury by mitigating mitochondrial oxidative stress and enhancing mitochondrial metabolism 48. Additionally, the normalization of mitochondrial response sensitivity and mitochondrial senescence through the activation of mitochondrial biogenesis was demonstrated in a mouse model of cardiac hypertrophy 49. In this study, we observed the pathological alterations and protective mechanisms associated with TRZ-mediated mitochondrial biogenesis in cardiomyopathy. Our findings contribute additional evidence supporting the cardioprotective properties of mitochondrial biogenesis in cardiovascular disease.

AMP-activated protein kinase (AMPK) is a highly conserved serine/threonine protein kinase, playing a pivotal role in the regulation of biological energy metabolism. Activated AMPK serves as a monitor for mitochondrial function and cellular energy status 50. At the molecular level, PGC-1α transcription and activity are regulated by the AMPK pathway 51. The protective function of AMPK against diabetic cardiomyopathy, myocardial ischemia-reperfusion injury, cardiac remodeling, and inflammation-associated myocardial injury has been extensively studied 52. Given the beneficial effects of AMPK and PGC-1α on mitochondrial homeostasis and cardioprotection, it is crucial to assess their impact on trastuzumab-mediated myocardial injury and mitochondrial damage. Research has demonstrated that overexpression of SIRT3 activates the AMPK pathway, thereby ameliorating mitochondrial biogenesis. This process is essential for maintaining mitochondrial redox homeostasis, sustaining mitochondrial respiration, and inhibiting mitochondrial apoptosis 53. In our study, SIRT3 knockout mice exhibited no significant changes in mitochondrial biosynthesis pathway proteins such as PGC-1α, NRF-1, NRF-2, etc. Notably, administration of TRZ stimulation to SIRT3 knockout mice resulted in a significant increase in cardiac scorched death, an intensified inflammatory response, a significant enhancement of fibrosis, a notable decrease in mitochondrial biosynthesis, and the inhibition of the AMPK pathway. It is evident that in the clinical use of trastuzumab, patients with underlying diseases, particularly cardiac and metabolic conditions, are at a higher risk of developing cardiotoxic effects. Thus, our study holds significant clinical relevance in understanding the cardiotoxic effects of TRZ. Furthermore, it offers a novel therapeutic target and insight for mitigating TRZ-induced cardio-oncology treatment.

Polyamines constitute a class of small-molecule compounds, encompassing spermine, spermidine, and putrescine, and are ubiquitously present in prokaryotic and eukaryotic tissue cells. These small-molecule aliphatic amine families play diverse biological roles, including anti-inflammatory, antioxidant, anti-apoptotic functions, DNA damage repair, enhancement of autophagy, and promotion of cell proliferation, among others. The literature reports highlight the significant role of polyamines in various pathophysiological processes, including cardiac hypertrophy, myocardial ischemia, diabetes, pancreatitis, and tumors. Literature reports demonstrate that exogenous spermidine inhibits ischemia/reperfusion-induced mPTP opening by scavenging free radicals. Moreover, exogenous spermidine retards cardiac aging in rats by fostering mitochondrial biosynthesis 30. In our study, supplementation of exogenous spermine attenuated TRZ-mediated expression of GSDMD, GSDME, IL-1β, Pro-IL-18, Pro-Caspase-1, Cleaved-Caspase-9, and NLRP3 in mice hearts. This indicates that exogenous spermidine inhibits the trastuzumab-mediated release of inflammatory factors, mitigates cardiomyocyte charring, and enhances cardiac function. Additionally, we investigated whether exogenous spermidine plays a role in enhancing mitochondrial function through the promotion of mitochondrial generation and the maintenance of mitochondrial quality control. The findings revealed that exogenous spermidine markedly attenuated the TRZ-induced decrease in the expression of mitochondrial biosynthesis pathway proteins, including PGC-1α, NRF-1, NRF-1, and TFAM. Additionally, exogenous spermidine reduced the expression of DRP1, increased the expression of OPA1. Notably, the addition of the SPD inhibitor, DFMO, nullified the effects of SPD. Previous studies have demonstrated that spermidine can induce mtROS-dependent AMPK activation 54.

Considering the proliferation-promoting and cytoprotective effects of polyamines on cultured human cancer cells or xenografted human tumors developed in immunodeficient mice, polyamines may exhibit procarcinogenic properties. In contrast to the potential procarcinogenicity associated with polyamines, supplementation with spermidine reduces tumorigenesis in mice and exerts anticancer effects 55. Dietary supplementation with spermidine diminished the severity of hepatic fibrosis and the incidence of chemical damage-induced hepatocellular carcinoma in mice. Additionally, spermidine attenuated the growth of CT26 colorectal tumors transplanted into immunocompetent mice 56. Augmenting dietary polyamine intake (via foods rich in spermidine, spermine, and putrescine) postponed chemically induced tumorigenesis in young BALB/c male mice, notwithstanding an increase in maximum tumor size 57. Spermidine supplementation diminished the growth of transplantable tumors in chemotherapeutic mice. This effect of spermidine is also observed in fasting or low-calorie diets 58 and is mediated by the stimulation of immune surveillance. Therefore, spermine augments the anticancer immune response by depleting immunosuppressive cells, such as regulatory T lymphocytes (Treg cells), in the tumor bed 59. Tumors grown in mice lacking cytotoxic T lymphocytes do not exhibit a reduction in their growth in response to spermidine 59. Besides its impact on adaptive immunity, the production of spermine mediated by spermidine synthase seems to be a determinant of the antitumor effects of tumor-associated macrophages in the progression of colorectal cancer 56. These findings may provide an explanation for the chemopreventive effect of externally supplied spermidine in *vivo*.

In conclusion, we found that trastuzumab induces cardiomyocyte pyroptosis and impairs cardiac function, particularly in SIRT3 knockout mice, exacerbating the extent of TRZ-induced injury. Additionally, supplementation with exogenous spermidine mitigated TRZ-mediated cardiac injury, offering a potential new target for the treatment of TRZ-induced cardiotoxicity. These findings establish an experimental foundation for clinical applications in TRZ-mediated tumor cardiology treatment and highlight the significant therapeutic potential of exogenous spermidine.

## Supplementary Material

Supplementary figures.

## Figures and Tables

**Table 1:** List of antibodies used in this study.

Antibodies	Supplier	Product code
NRF-1	CST	46743S
TFAM	Abcam	Ab176558
PGC-1α	Abcam	Ab191838
UQCRC2	Proteintech	67547-1-Ig
SDHB	Proteintech	67600-1-Ig
COXIV	Proteintech	11242-1-AP
NDVFB8	Abcam	Ab251160
ATP5A1	Proteintech	14676-1-AP
NRF-2	CST	12721S
GSDME	CST	40618S
Cleaved-Caspase-9	CST	9505P; 7237S
IL-1β	CST	12703S
Caspase-1	CST	2225S
Pro-IL-18	Proteintech	10663-1-AP
SIRT3	CST	2627S
GSDMD	CST	39754S
NLRP3	CST	15101S
COL1	Proteintech	14695-1-AP
COL3	Proteinrech	22734-1-AP
α-SMA	SAB	#41550
DRP1	Proteintech	12957-1-AP
PI3K	Abcam	Ab182651
p-PI3K	CST	17366S
AKT	CST	4691S
p-AKT	CST	4060S
OPA1	Proteintech	27733-1-AP
AMPK	CST	5831S
p-AMPK	CST	2535S
ErbB2	CST	2165S
CAT	CST	14097S
ULK1	Proteintech	20986-1-AP

**Table 2:** Primer sequences used for RT-qPCR.

IL-1β-F	5'-GGCTTCCTTGTGCAAGTGTC-3'
IL-1β-R	5'-TGTCGAGATGCTGCTGTGAG-3'
PGC-1α-F	5'-GTGCAGCCAAGACTCTGTATGG-3'
PGC-1α-R	5'-GTCCAGGTCATTCACATCAAGTTC-3'
NRF-1-F	5'-TTACTCTGCTGTGGCTGATGG-3'
NRF-1-R	5'-CCTCTGATGCTTGCGTCGTCT-3'
TFAM-F	5'-AAATGGCTGAAGTTGGGCGAAGTG-3'
TFAM-R	5'-AGCTTCTTGTGCCCAATCCCAATG-3'
β-actin-F	5'-TCGTGCGTGACATTAAAGAG-3'
β-actin-R	5'-ATTGCCGATAGTGATGACCT-3'

**Table 3:** Primer sequences used for siRNA PGC-1α.

PGC-1α-558-F	5'-GAGAAUUCAUGGAGCAAUA-3'
PGC-1α-558-R	5'-UAUUGCUCCAUGAAUUCUC-3'
PGC-1α-1436-F	5'-AGACAAGACCAGUGAACUA-3'
PGC-1α-1436-R	5'-UAGUUCACUGGUCUUGUCU-3'
PGC-1α-2001-F	5'-AGGCAGAAGCAGAAAGCAA-3'
PGC-1α-2001-R	5'-UUGCUUUCUGCUUCUGCCU-3'
