# Supplementary material for: Exogenous SPD inhibits trastuzumab-mediated cardiomyocyte pyroptosis through SIRT3-regulated mitochondrial quality control
Source: Int J Biol Sci. 2025 Jun 9;21(9):4027–50. doi: 10.7150/ijbs.110580 (PMC12223783; doi:10.7150/ijbs.110580)
Supplement: Supplementary file 1 — Supplementary figures. [file ijbsv21p4027s1.pdf]

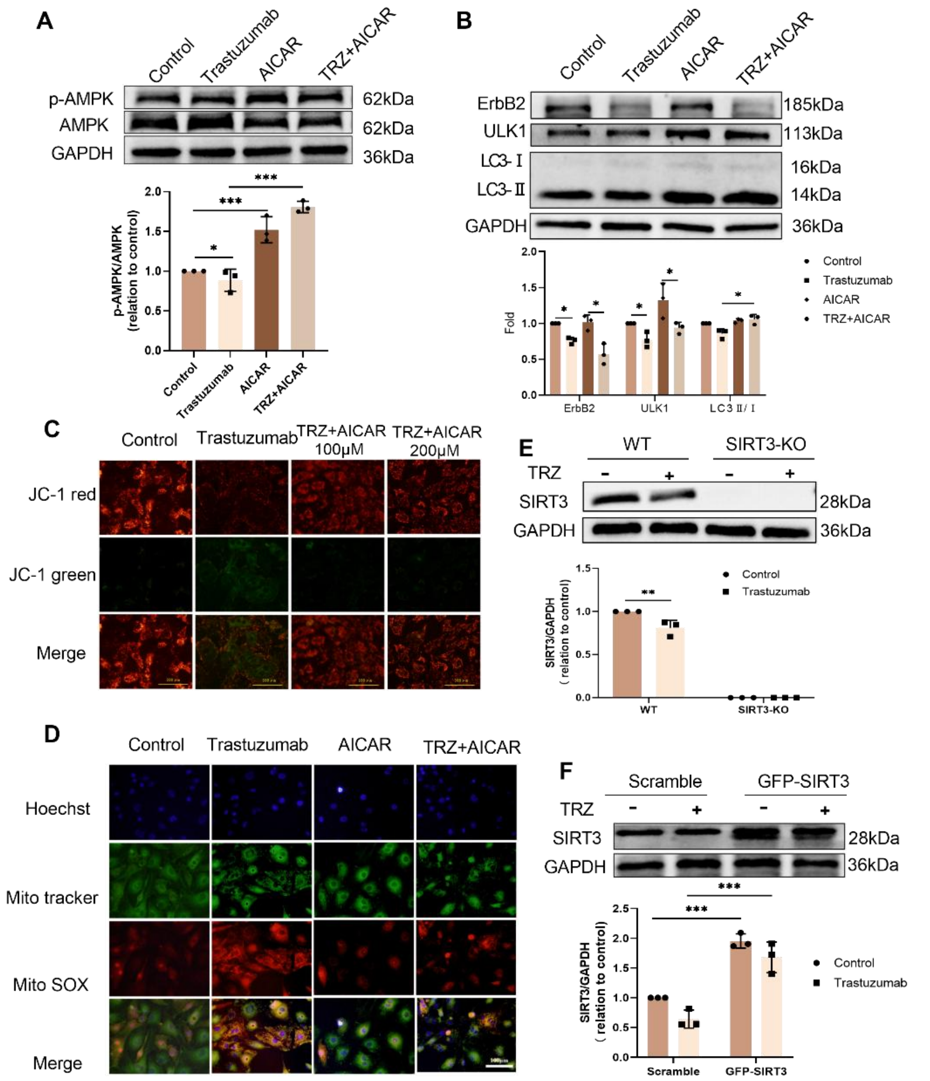

Supplementary Fig. 1 AMPK agonist AICAR treatment attenuates TRZ-induced decrease in cardiomyocyte autophagy, altered membrane potential, and enhanced mitochondrial oxidative stress (A) Protein expression levels of p-AMPK were analyzed by western blotting after TRZ and AICAR treatment in HL-1 cardiomyocytes. Data are expressed as mean ± SD (n = 3). **P < 0.05, ***P < 0.001. ANOVA*. (B) Protein expression levels of ErbB2, ULK1, and LC3-II/I were analyzed by western blotting after TRZ and AICAR treatment in HL-1 cardiomyocytes. Data are expressed as mean ± SD (n = 3). **P < 0.05. ANOVA*. (C) JC-1 fluorescent probe to detect changes in mitochondrial membrane potential after TRZ and different concentrations of AICAR treatments

in primary lactating rat cardiomyocytes, scale bar = 100 μm. (D) Mito SOX fluorescent probe to detect mitochondrial reactive oxygen species accumulation after TRZ and AICAR treatments in primary lactating rat cardiomyocytes. Scale bar = 100 μm. (E) Protein expression levels of SIRT3 in WT and SIRT3-KO mice were analyzed by western blotting. Data are expressed as mean ± SD (n = 3). $^{**}P < 0.01$. *ANOVA*. (F) Western blotting assay to analyze the protein expression level of SIRT3 in HL-1 cardiomyocytes. Data are expressed as mean ± SD (n = 3). $^{***}P < 0.001$. *ANOVA*.

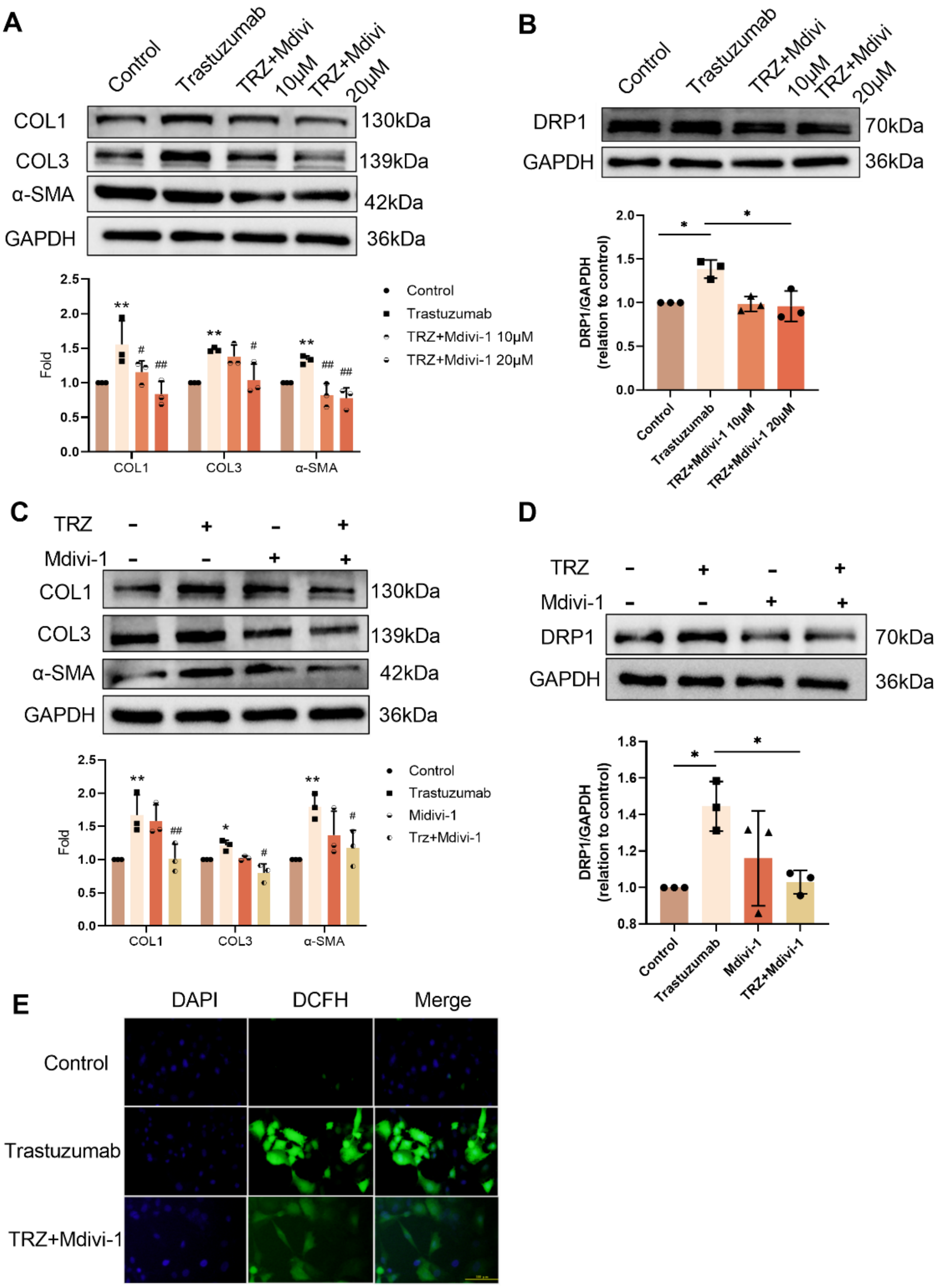

Supplementary Fig. 2 Mitochondrial division inhibitor Mdivi-1 treatment inhibits TRZ-induced fibrosis in primary fibroblasts (A) Protein expression levels of fibrotic proteins COL1, COL3 and α-SMA were analyzed by western blotting in primary fibroblasts after treatment with TRZ and different concentrations of the mitochondrial division inhibitor Mdivi-1. Data are expressed as mean ± SD (n = 3). Compared with control group, ***P < 0.01. ANOVA*. Compared

with Trastuzumab group, *#P < 0.05, ##P < 0.01. ANOVA*. (B) Protein expression levels of DRP1 were analyzed by western blotting in primary fibroblasts after treatment with TRZ and different concentrations of the mitochondrial division inhibitor Mdivi-1. Data are expressed as mean ± SD (n = 3). **P < 0.05. ANOVA*. (C) Protein expression levels of fibrotic proteins COL1, COL3 and α-SMA were analyzed by western blotting after TRZ and Mdivi-1 treatment of cells. Data are expressed as mean ± SD (n = 3). Compared with the control group, **P < 0.05, **P < 0.01. ANOVA*. Compared with the Trastuzumab group, *#P < 0.05, ##P < 0.01. ANOVA*. (D) Protein expression levels of the fibrotic protein DRP1 were analyzed by western blotting method after TRZ and Mdivi-1 treatment of cells. Data are expressed as mean ± SD (n = 3). **P < 0.05. ANOVA*. (E) DCFH fluorescent probe to detect reactive oxygen species content in primary fibroblasts, scale bar = 100 μm.

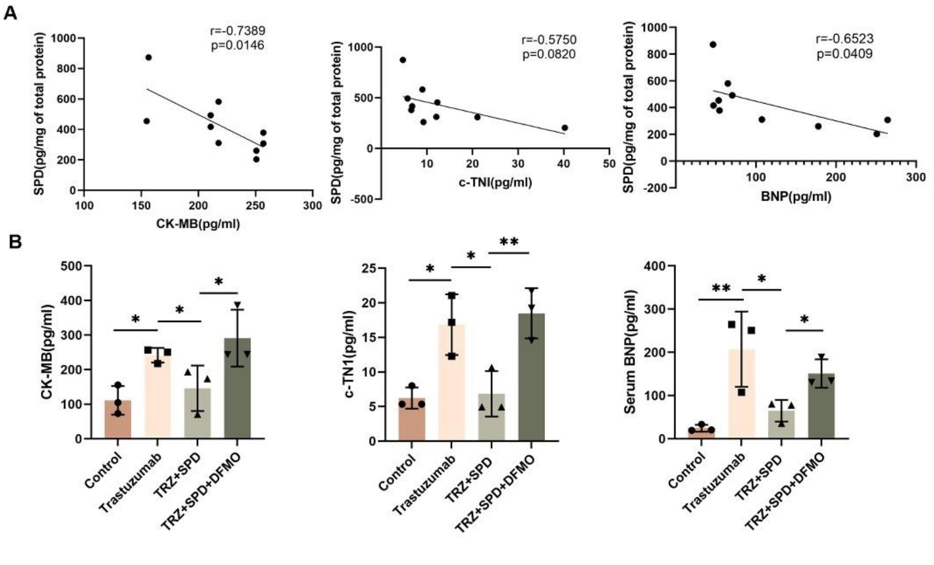

Supplementary Fig. 3 SPD was inversely associated with cardiac injury markers (A)Correlation analysis of SPD and myocardial injury markers CK-MB, c-TNI and BNP in mouse serum after TRZ treatment (n=10). (B) The expression levels of myocardial injury markers CK-MB, c-TNI and BNP in mouse serum. Mean ± SD (n = 3). *P < 0.05. **P < 0.01. ANOVA.

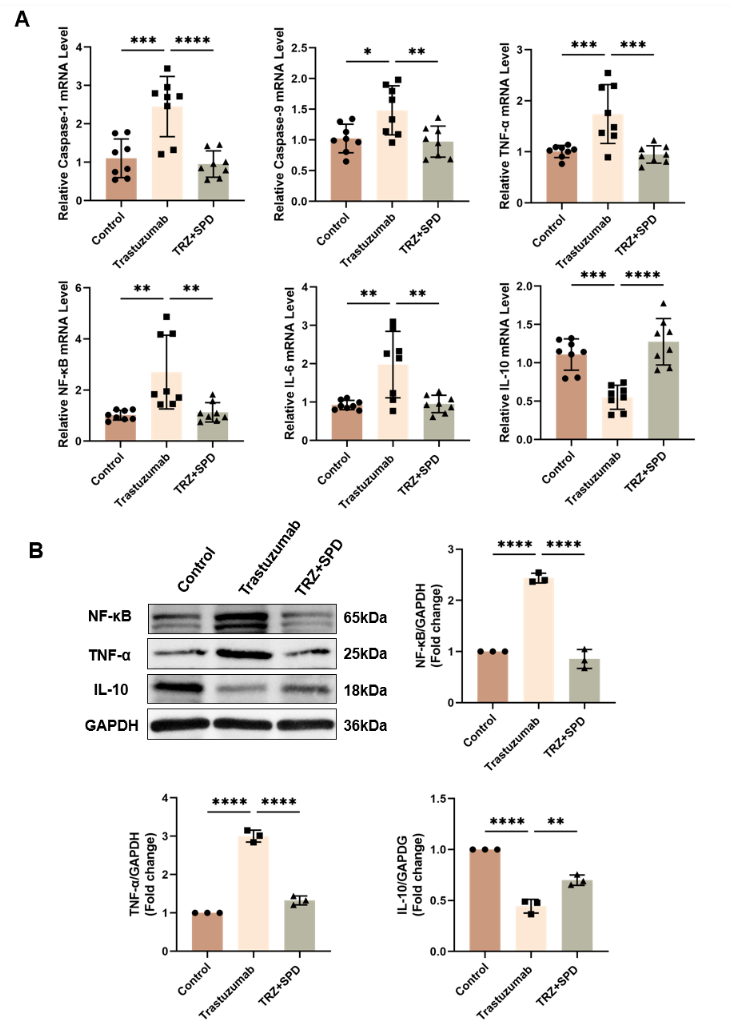

Supplementary Fig. 4 Exogenous SPD can inhibit the increase in myocardial inflammatory response induced by TRZ (A) qPCR was performed to detect the mRNA expression levels of Caspase-1, Caspase-9, TNF-α, NF-κB， IL-6 and IL-10 in the mice myocardial tissue. Mean ± SD (n=8). **P < 0.05, **P < 0.01, ***P < 0.001, and ****P < 0.0001. ANOVA.* (B) Western blotting was performed to analyze the protein expression levels of the NF-κB, TNF-α and IL-10, in the mice myocardial tissues. Mean ± SD (n = 3). ***P < 0.01, ***P < 0.001 ****P < 0.0001. ANOVA.*
